# Fluorogenic Trp(redBODIPY) cyclopeptide targeting keratin 1 for imaging of aggressive carcinomas†

**DOI:** 10.1039/c9sc05558d

**Published:** 2019-12-18

**Authors:** Ramon Subiros-Funosas, Vivian Cheuk Lam Ho, Nicole D. Barth, Lorena Mendive-Tapia, Morena Pappalardo, Xavier Barril, Ruoyu Ma, Cheng-Bin Zhang, Bin-Zhi Qian, Miquel Sintes, Ouldouz Ghashghaei, Rodolfo Lavilla, Marc Vendrell

**Affiliations:** Centre for Inflammation Research, University of Edinburgh 47 Little France Crescent EH16 4TJ Edinburgh UK marc.vendrell@ed.ac.uk; Laboratory of Physical Chemistry, Facultat de Farmàcia, Universitat de Barcelona and Institut de Biomedicina de la Universitat de Barcelona (IBUB) Av. Joan XXIII s/n 08028 Barcelona Spain; MRC Centre for Reproductive Health, University of Edinburgh 47 Little France Crescent EH16 4TJ Edinburgh UK; Laboratory of Medicinal Chemistry, Faculty of Pharmacy, University of Barcelona and Institut de Biomedicina de la Universitat de Barcelona (IBUB) Avda Joan XXIII 27-30 Barcelona 08028 Spain rlavilla@ub.edu

## Abstract

Keratin 1 (KRT1) is overexpressed in squamous carcinomas and associated with aggressive pathologies in breast cancer. Herein we report the design and preparation of the first Trp-based red fluorogenic amino acid, which is synthetically accessible in a few steps and displays excellent photophysical properties, and its application in a minimally-disruptive labelling strategy to prepare a new fluorogenic cyclopeptide for imaging of KRT1+ cells in whole intact tumour tissues.

## Abstract 2

Trp(redBODIPY) is the first red-emitting Trp-based amino acid for the preparation of fluorogenic peptides with retention of target binding affinity.

## Graphical abstract

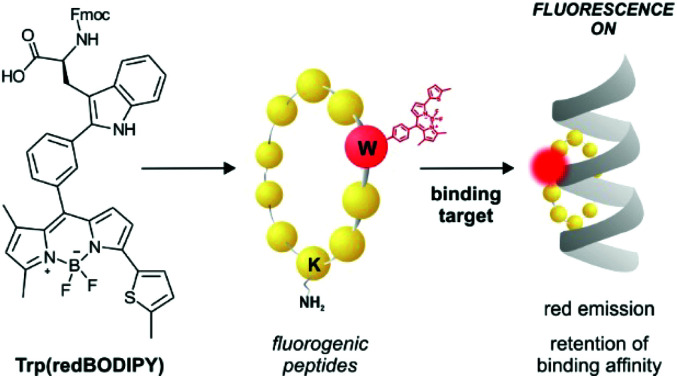

## Introduction

In recent years, optical molecular imaging has undergone substantial development into the direct visualisation of cellular features that are associated with disease processes.^1–6^ In this context, the molecular complexity of the probes needed for optimal biological performance requires modern synthetic methods, often beyond standard protocols.^7^ Our group and others have demonstrated the value of peptide-based optical probes as powerful tools to monitor cellular events in real time.^8–10^ Suitable derivatisation of peptides with fluorophores is of paramount importance in chemical biology and optical imaging. The most conventional labelling strategies involve the formation of amide bonds between amino groups at the N-terminal end or at the side chains of lysine (Lys) residues. However, in some sequences this approach alters important features of the native peptides (*e.g.*, net charge, H-bonding pattern) and can compromise the recognition and binding affinity for their biomolecular targets.

Alternatively, fluorescent amino acids have emerged as minimally-disruptive labelling strategy to construct optical peptide-based probes.^11–14^ Our group has developed efficient protocols to couple green-emitting BODIPY chromophores to the C_2_ position of tryptophan (Trp) using Pd-catalysed C–H activation processes.^15–17^ However, a major shortcoming of existing fluorogenic amino acids is their fluorescence emission in blue or green regions of the visible spectrum, which offer limited sensitivity due to tissue autofluorescence, particularly during tissue imaging. Herein we report the first red-emitting Trp-based fluorogenic amino acid and its optimal incorporation into a cyclic peptide to image the protein keratin 1 (KRT1), which is overexpressed in aggressive breast cancer tumours.

Breast cancer is one of the most common malignancies in women, accounting for 15% of total female cancer death in the UK and making it the third most common cause of cancer death. Early diagnosis is critical to achieve effective therapeutic outcomes in patients and to enhance the survival rates.^18^ Tomographic and mammography imaging techniques, together with magnetic resonance imaging, are regularly used in the clinic to enhance the accuracy of diagnostic readouts, although they are hampered by their limited spatial resolution and poor specificity at the molecular level.^19–21^ To address these shortcomings, optical molecular imaging is being increasingly used into routine clinical practice, and some translational probes to study biological systems *in situ* have been reported.^22,23^

**Fig. 1:**
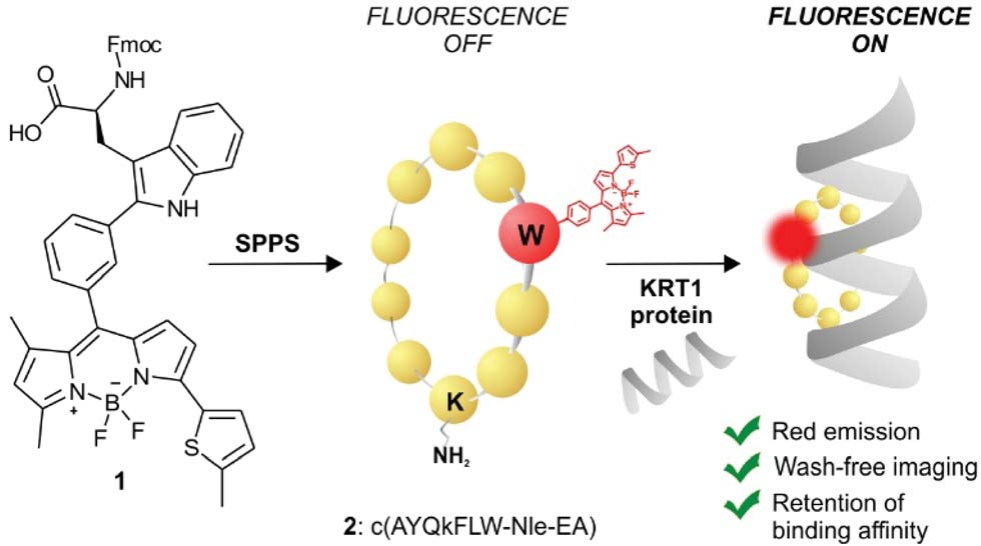
Schematic illustration of the first red fluorescent Trp-based amino acid and into incorporation into cyclic peptides to produce fluorogenic probes for imaging of KRT1+ aggressive cancer cells.

KRT1 is focally expressed in squamous carcinomas, which is associated with a rare and aggressive pathology within breast cancer.^24,25^ KRT1 is overexpressed in some breast cancer cell lines (*e.g.*, MDA-MB-231 and MDA-MB-435) whereas is only present in low levels in non-cancerous cells (*e.g.*, HUVEC).^26,27^ The group of Kaur identified by phage display the linear peptide sequence p160, a 12-mer that can strongly bind KRT1 with *K*_d_ values around 1 μM.^26^ In subsequent optimised generations, the authors designed a proteolytically-stable cyclic derivative including a d-Lys (Fig. 1).^27^ We envisaged that red fluorogenic analogues of the KRT1-binding sequence would enable imaging of aggressive breast carcinomas, hence we designed a Trp(redBODIPY) building block (**1**, Fig. 1) for minimally-invasive labelling of peptides with a fluorophore emitting in the red region. Furthermore, we compared the performance of this new methodology to conventional labelling approaches and prepared an analogue of peptide **2** by coupling the commercial red fluorescent BODIPY TR to the amino group on the side chain of d-Lys.

## Results and discussion

### Design and synthesis of Trp(redBODIPY)

**Scheme 1:**
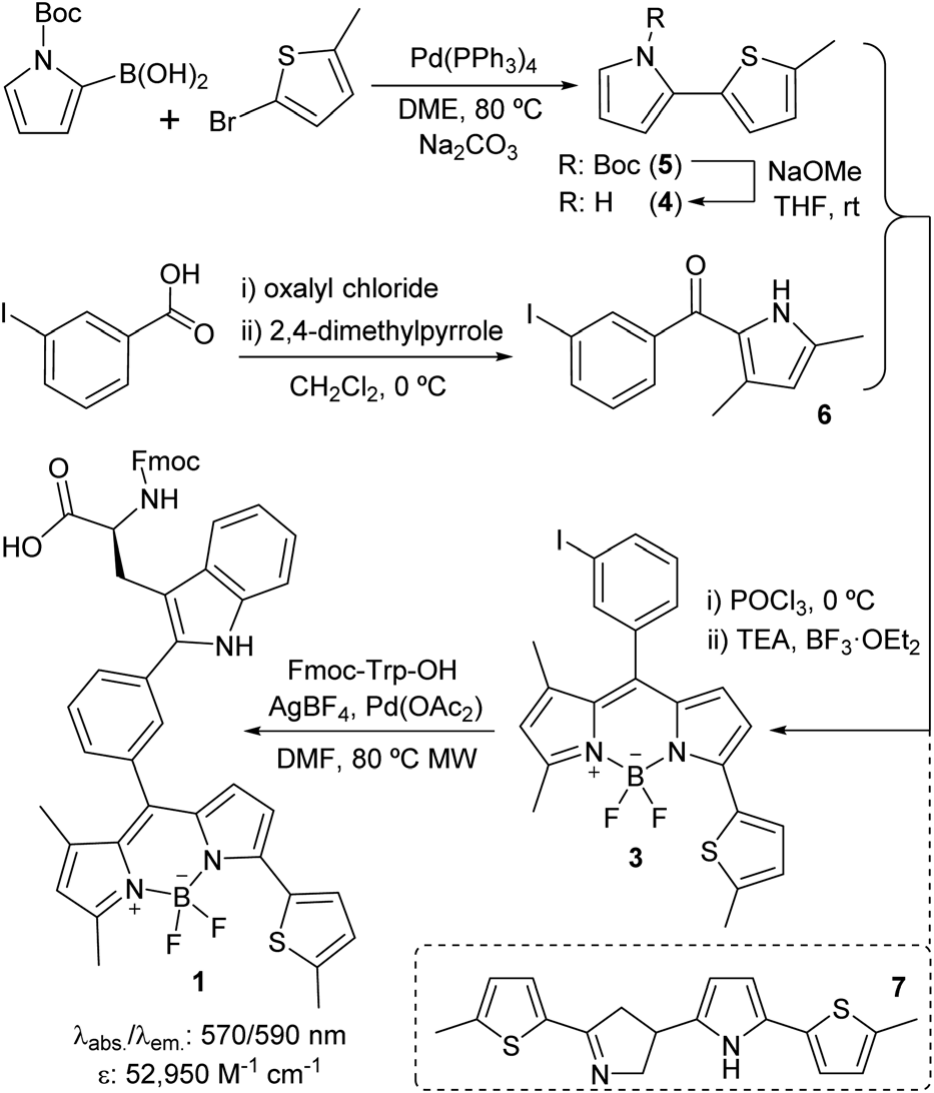
Synthetic scheme for the preparation of the Fmoc-protected Trp(redBODIPY) (**1**) building block for SPPS.

We designed Trp(redBODIPY) (**1**) by extending the electronic conjugation of Trp-BODIPY with a 2-methylthiophene moiety directly coupled to the position 3 of the BODIPY scaffold. As in other BODIPY dyes,^28–30^ we envisaged that this small (<100 Da) chemical modification would significantly red-shift the fluorescence emission wavelength while retaining most of the molecular recognition properties derived from the Trp-based amino acid. To this end, we envisaged a synthetic approach for the amino acid **1** in which the key step would involve direct arylation of Trp using Pd-catalysed C–H activation^31^ with an appropriate haloaryl BODIPY derivative (Scheme 1).

We prepared the 3-iodophenyl-BODIPY precursor **3** through a convergent approach, suitable for the preparation of non-symmetric BODIPY dyes, *via* regioselective synthesis of the thiophenepyrrole derivative **4**. Compound **4** was synthesized by Suzuki–Miyaura coupling between the commercially available *N*-Boc-pyrrole-2-boronic acid and 2-bromo-5-methylthiophene, followed by basic deprotection. On the other hand, the Friedel–Crafts acylation between the *m*-iodobenzoic acid chloride and 2,4-dimethylpyrrole conveniently afforded compound **6**. The condensation of compounds **4** and **6***via* POCl_3_ activation furnished 3-iodophenyl-BODIPY **3** after subsequent coordination with BF_3_·OEt_2_, although in low yields (22%).^32^ Attempts to increase the recovery yields by altering the condensation reaction conditions, including the presence of base or other acid catalysts, were unsuccessful. Although literature precedents account for the feasibility of this synthetic protocol,^33–35^ in this case we observed the formation of the main byproduct **7** as a result of the competitive dimerisation of the thiophenepyrrole **5** due to the acid-catalysed interaction of an electron-rich pyrrole relatively free of steric hindrance.^36,37^ Finally, we performed Pd-catalysed C–H arylation of compound **3** with Fmoc-Trp-OH to obtain the Fmoc-protected Trp(redBODIPY) (**1**), ready for use in solid-phase peptide synthesis (SPPS). Spectral characterisation of Trp(redBODIPY) (**1**) confirmed notable fluorogenic behaviour with bathochromic shifts in absorbance and emission wavelengths (*i.e.*, ∼60 nm longer when compared to Trp-BODIPY) and fluorescence spectrum covering the 570–610 nm range (Fig. S1–S4†).

### Synthesis and characterisation of fluorescent cyclopeptides

**Scheme 2:**
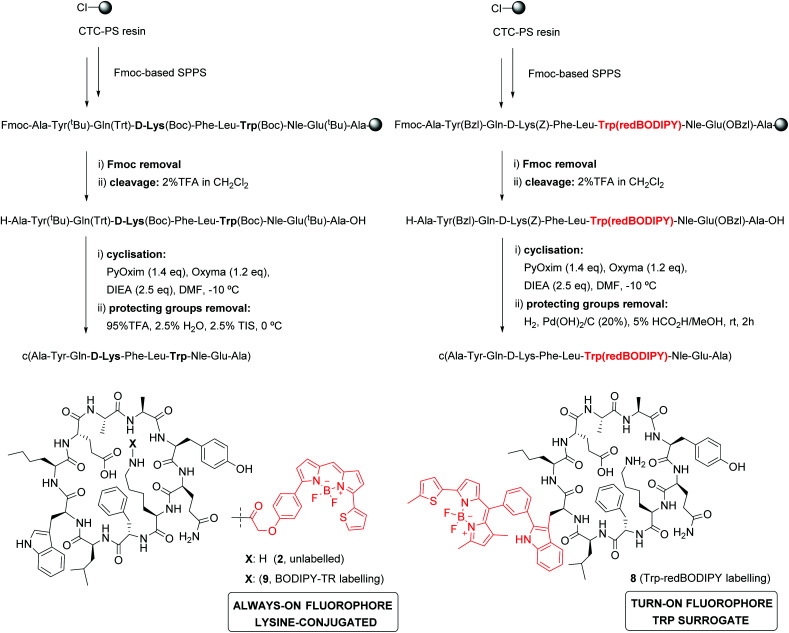
Synthesis of KRT1-binding peptides. A combined solid- and solution-phase synthetic approach was employed to obtain unlabelled (**2**), Trp(redBODIPY)-labelled (**8**) and BODIPY TR-labelled (**9**) peptides.

In view of the suitable optical properties of Trp(redBODIPY) (**1**), we proceeded to the SPPS of fluorescent derivatives of the KRT1-binding cyclopeptide **2**. Syntheses were performed on a 2-chlorotrityl chloride polystyrene resin using standard deprotection and coupling SPPS conditions (*i.e.*, DIC and Oxyma).^38^ Building blocks for polar amino acids were protected with H_2_-labile groups (*i.e.*, Tyr(Bzl), Glu(OBzl), d-Lys(Z)) that are compatible with the BODIPY structure.^39^ In addition to the unlabelled cyclopeptide **2**, we prepared the Trp(redBODIPY)-labelled counterpart (**8**) and the fluorescent peptide including the commercial dye BODIPY TR, with similar absorbance/emission wavelengths to the amino acid **1** (Fig. S5†), which was coupled to the side chain of the d-Lys (**9**). All peptides were obtained in a similar manner, combining an initial solid-phase sequence elongation followed by head-to-tail peptide cyclisation and final removal of the protecting groups in solution (Scheme 2).

Notably, we did not observe any signs decomposition of Trp(redBODIPY) in any of the solid-phase procedures, highlighting the chemical stability and suitability of the amino acid for peptide synthesis. The fluorescent peptide **9** was readily obtained by formation of an amide bond between the amino-containing peptide **2** and the activated succinimidyl ester of the commercially available dye BODIPY TR. The three peptides were purified by preparative HPLC and isolated in purities >95% (full characterisation data in ESI†).

**Fig. 2:**
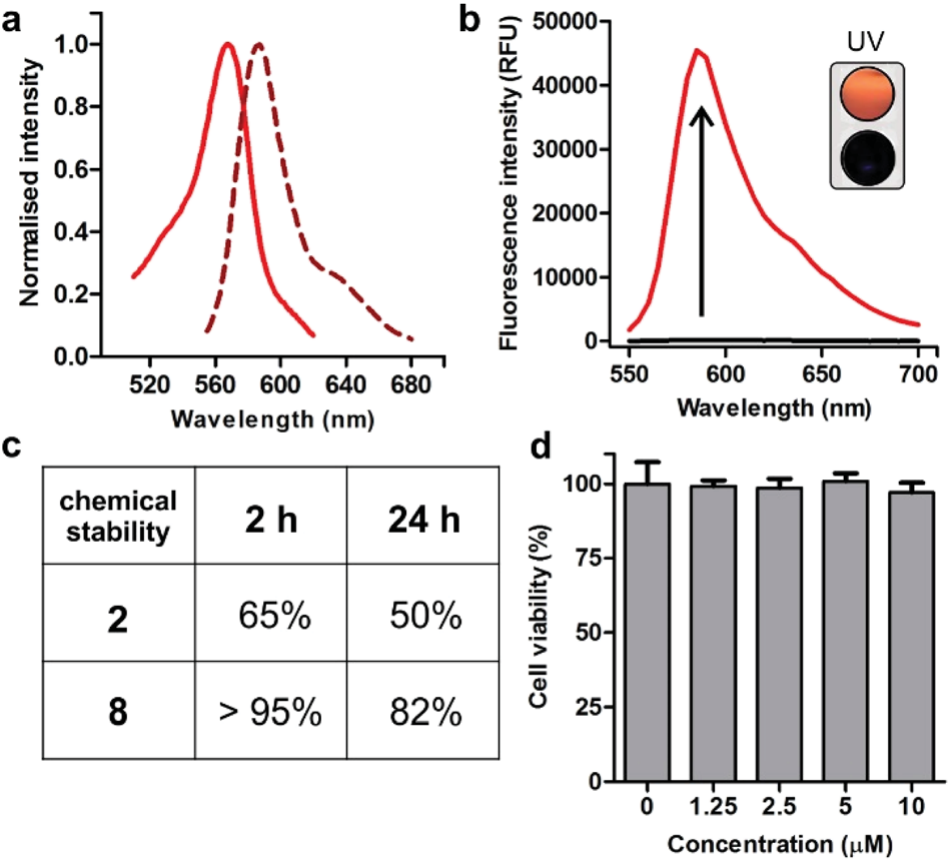
(a) Absorbance and emission spectra of Trp(redBODIPY)-labelled peptide **8** (10 μM) in EtOH. Normalised absorbance (solid line) and fluorescence emission (dashed line, *λ*_exc._: 520 nm). (b) Fluorogenic behaviour of cyclopeptide **8** (40 μM) in hydrophilic and hydrophobic media (70-fold). Peptide **8** was incubated in PBS (black) or in phosphatidylcholine liposome suspensions (red, *λ*_exc._: 520 nm). (Inset) Naked eye pictograms of both solutions under a hand-held UV lamp using 365 nm excitation. (c) Stability of peptides in a protease cocktail by HPLC monitoring. (d) Viability of HeLa cells after incubation with different concentrations of peptide **8**. Values are presented as means ± s.d. (*n* = 4). Non-significant differences (*p* > 0.05) were observed between the untreated control and any of the treatments.

Spectroscopic examination of the cyclopeptide **8** showed that it retained the optical characteristics of Trp(redBODIPY) (**1**) with fluorescence emission around 600 nm, where tissue autofluorescence is typically low (Fig. 2). Peptide **8** also displayed remarkable fluorogenic behaviour, with low emission in aqueous media and up to 70-fold fluorescent increase in hydrophobic microenvironments (*i.e.*, phosphatidylcholine liposome suspensions) for enhanced signal-to-noise ratios (Fig. 2). Given that proteases are abundant enzymes at tumour sites, we also investigated the potential degradation of the cyclopeptides **2** (unlabelled) and **8** [Trp(redBODIPY)-labelled] when subjected to strong proteolytic environments. We incubated both peptides with a commercially available protease cocktail at 37 °C and recorded their chemical stability by HPLC-MS over time. As expected for cyclic structures, both peptides displayed good resistance to proteolytic degradation, with the main products (**2** and **8**) being the most abundant species even after 24 h in the proteolytic cocktail (Fig. 2 and S6†). We also observed that the incorporation of the amino acid Trp(redBODIPY) into the structure of the cyclopeptide **2** slightly augmented its resistance to proteases, likely due to the inherent properties of unnatural amino acids. Moreover, the incorporation of Trp(redBODIPY) did not confer any cytotoxicity to the KRT1-binding sequence as determined in cell viability assays with HeLa cells (Fig. 2).

### Computational analysis

**Fig. 3:**
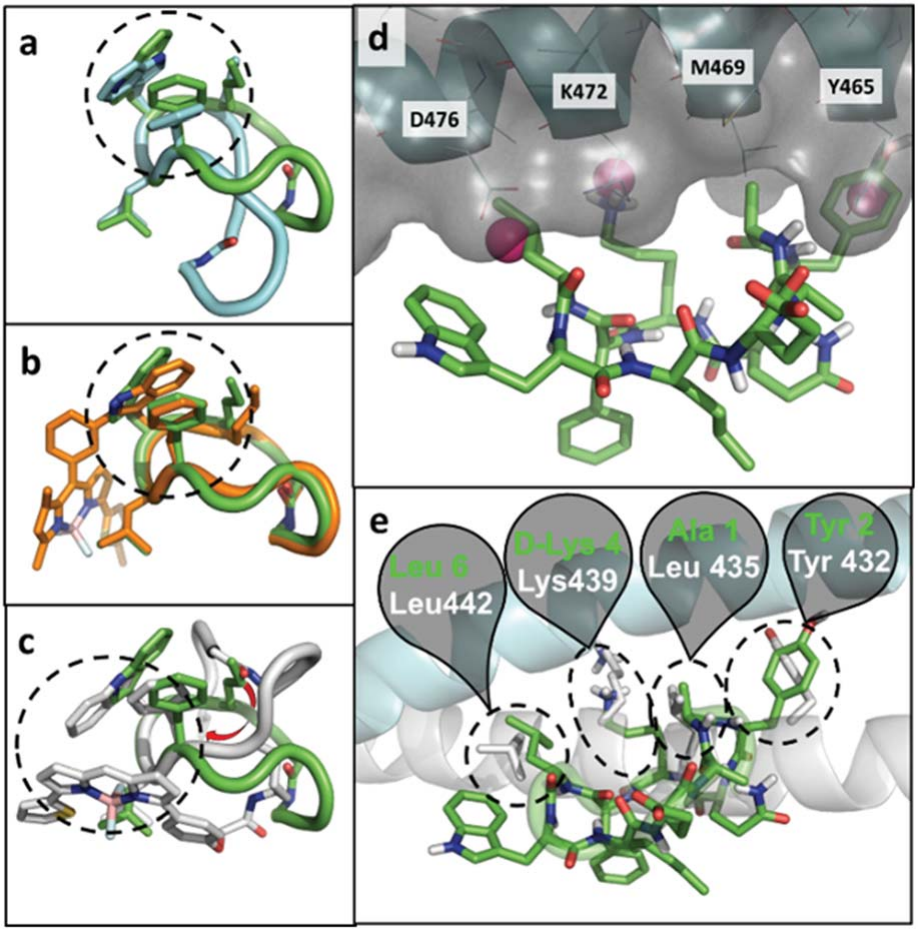
Representative conformations of unlabelled peptide **2** (green), overlapped to: (a) the second most populated conformation of **2** (cyan); (b) the most representative conformation of peptide **8** (orange); and (c) the most representative conformation of peptide **9** (grey). The dashed circles highlight the hydrophobic core on each structure. (d) Putative binding mode of peptide **2** (green) bound to KRT1 (grey surface with cyan cartoon). Some contact residues are labelled for reference. The pink spheres indicate binding hot spots identified with MDmix. (e) Overlap with the experimental binding mode of KRT10 (grey cartoon and sticks), indicating the residues of the peptide **2** (green) and protein (white) that coincide. In this cartoon, KRT1 is shown in cyan.

At the computational level, we investigated the effects of BODIPY labelling on the conformational preferences of the cyclopeptide **2** and on its binding to KRT1. For the first aspect, we used all-atom molecular dynamics simulations in explicit aqueous solvation. The peptidic backbone of the unlabelled peptide **2** was in rapid exchange among various conformations, but the two most populated ones (*i.e.*, representing 68% and 11% of the total population) were defined by the hydrophobic collapse of the side chains Trp, Phe and norleucine (Nle) (Fig. 3a). Trp(redBODIPY)-labelling preserved this hydrophobic core, and the most abundant conformation of peptide **8** (64% of the total population) clearly overlapped with the most abundant conformation of **2** (Fig. 3b). In contrast, BODIPY TR-conjugation on d-Lys disrupted the hydrophobic core and altered the conformational preferences of the macrocycle. In the most abundant conformation of peptide **9** (46% of total population), the side chain of Nle was no longer involved in the hydrophobic core. Instead, the aromatic rings of the BODIPY TR fluorophore stacked over the side chains of Phe and Trp, creating an alternative hydrophobic core. The labelling of d-Lys also increased the conformational diversity of peptide **9** (Table S1†), which could negatively affect its binding to KRT1. In contrast, the rigid Trp(redBODIPY) stabilised the original conformation of the unlabelled peptide **2** and reduced its accessible conformational space.

In order to further understand the impact of BODIPY labelling on the ability of the cyclopeptides to bind KRT1, we combined structural and sequence information of the protein to generate a putative binding mode (ESI† for full details). The confidence on the docking predictions was supported by the convergency of orthogonal data. The top-scoring docking solution for peptide **2** overlapped with multiple MDmix-predicted hot spots^40,41^ and produced an outstanding structural alignment with KRT10,^42^ the natural partner of KRT1 (Fig. 3d and e). The top-scoring solution of the Trp(redBODIPY)-labelled peptide **8** coincided with that of peptide **2**, whereas the lysine-modified peptide **9** bound differently and showed lower scores in the docking solutions (Fig. S12†). While this computational analysis does not take into consideration the complexity and potential heterogeneity of cellular systems (*e.g.* quaternary structures of KRT1 with other keratins), our results suggest that Trp(redBODIPY) labelling does not hinder the natural binding properties of compound **2**, in contrast to the BODIPY TR conjugation to d-Lys.

### Binding analysis and fluorescence live-cell imaging of KRT1+ cells

**Fig. 4:**
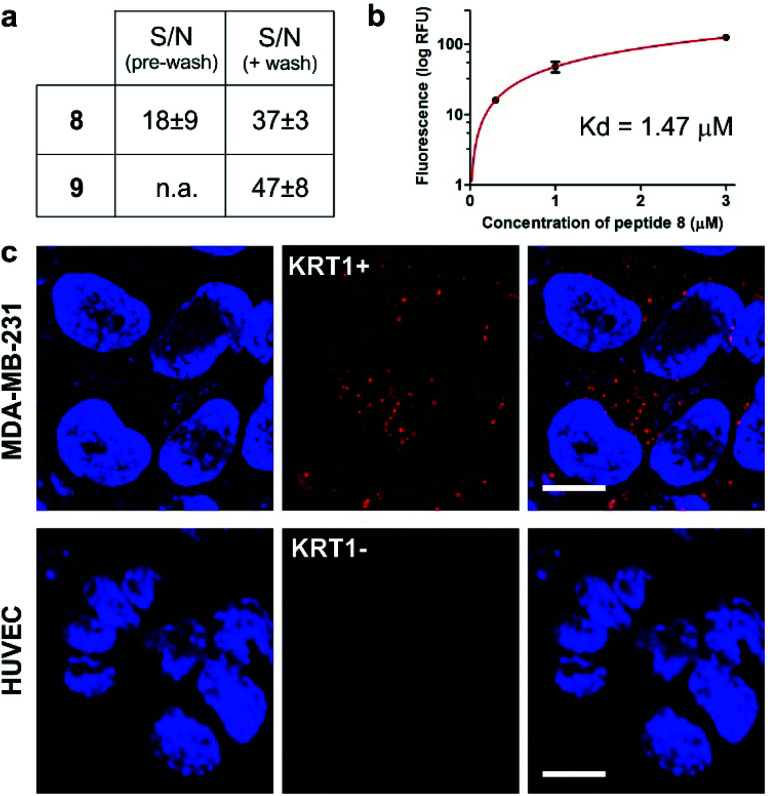
(a) Quantification of the signal-to-noise (S/N) ratios for peptides **8** and **9** in MDA-MB-231 cells before and after washing (fluorescence microscope images in Fig. S14†). Values are presented as means ± s.e.m. (*n* = 3). (b) Flow cytometric analysis of MDA-MB-231 (KRT1+ cells) upon incubation with increasing concentrations of the Trp(redBODIPY)-labelled peptide **8** (*R*^2^: 0.990). Values are presented as means ± s.d. (*n* = 3). (c) Fluorescence microscope images of cells expressing different levels of KRT1 (MDA-MB-231 as KRT1-positive, HUVEC as KRT1-negative cells) upon incubation with the peptide **8** (red, 1 μM) and Hoechst 33342 for nuclear counterstaining (blue). Scale bar: 10 μm.

We next examined the binding and imaging capabilities of the fluorescent cyclopeptides in human MDA-MB-231 cells as a KRT1-positive highly aggressive and invasive epithelial, triple-negative breast cancer cell line.^43^ First, we performed flow cytometric analysis to measure the relative binding affinity of Trp(redBODIPY)-labelled peptide **8** (Fig. 4) for KRT1-expressing cells. The *K*_d_ for peptide **8** was found to be around 1 μM, in agreement with the values previously reported for the unlabelled KRT1-binding sequences^26,27^ and in line with our results from the computational analysis. Notably, the analysis of peptide **9** in MDA-MB-231 cells indicated around 5-fold weaker binding profile (Fig. S13†), which indicates that the incorporation of a BODIPY-based amino acid at the hydrophobic site of cyclopeptide **2** is less perturbative than the classical derivatisation of a lysine residue with the BODIPY TR dye. We also compared the fluorogenic capabilities and signal-to-noise ratios of both peptides **8** and **9** by taking fluorescence microscopy images of MDA-MB-231 cells after incubation with the two peptides with and without washing. The fluorogenic character of Trp(redBODIPY) allowed us to acquire wash-free microscope images with good signal-to-noise ratios, whereas peptide **9** only discriminated subcellular structures after washing (Fig. 4 and S14†).

Finally, we used peptide **8** for live-cell imaging in both human MDA-MB-231 cells and HUVEC (Human Umbilical Vein Endothelial) cells, which have differential expression levels of KRT1. The red fluorogenic peptide **8** brightly stained MDA-MB-231 cells whereas it showed minimal staining of HUVEC cells under the same experimental conditions, indicating strong dependence on the expression levels of KRT1 (Fig. 4).

### Multi-colour imaging of KRT1+ cells and tumour-associated macrophages in aggressive tumours

Triple-negative breast cancer is an aggressive form of breast cancer with limited therapeutic options, and where new research tools for visualising and understanding its molecular basis are needed in order to develop more effective treatments. MDA-MB-231 cells are aggressive breast cancer cells, but there are limited tools to study how they interact with immune cells in the tumour microenvironment in whole intact tissues. Macrophages are the most abundant immune cells in tumours, and there is evidence that subpopulations of tumour-associated macrophages (TAMs) contribute to cancer progression and metastatic seeding.^44–48^ Therefore, we decided to examine whether the excellent properties of **8** as a red-emitting KRT1-binding peptide would enable multi-colour imaging of aggressive tumour cells in combination with TAM markers. First, we induced the tumour formation in NCI nude mice by orthotopic injection of MDA-MB-231 cells. Two weeks after, tumours were harvested for *ex vivo* multicolour fluorescence staining. In order to study the multicellular composition of aggressive tumour, we incubated tumour tissue sections with peptide **8** (red), anti-Iba1 as a TAM marker (green) and DAPI for nuclear staining (blue).

**Fig. 5:**
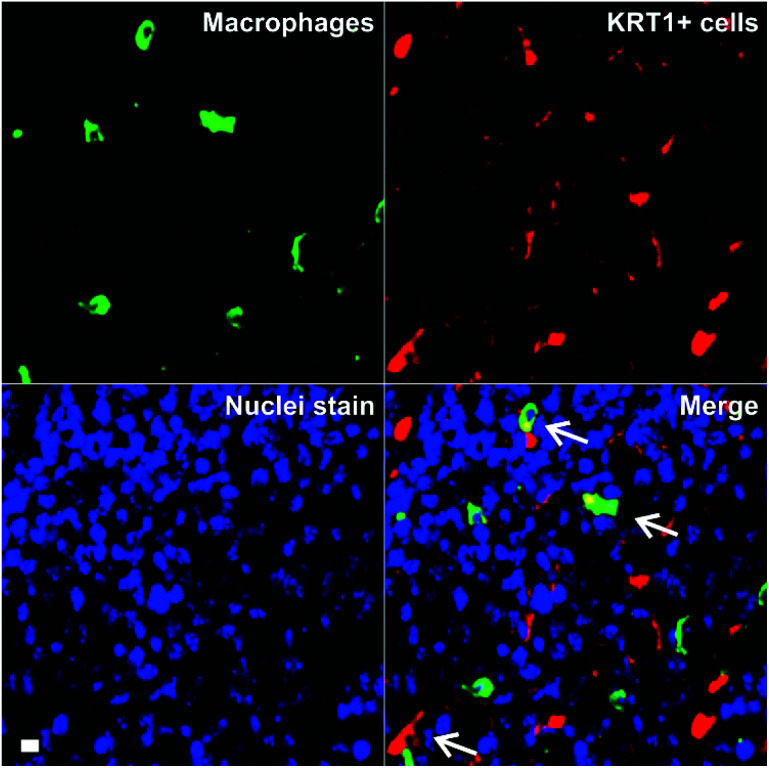
*Ex vivo* tissue imaging of aggressive carcinomas under multi-colour fluorescence confocal microscopy. Tumour-associated macrophages were stained in green by anti-Iba1 and KRT1+ cells were stained with peptide **8** (5 μM). DAPI was used for nuclear counterstaining (blue). TAM–cancer cell interactions are identified with white arrows. Scale bar: 10 μm.

As shown in Fig. 5, tumour tissues presented bright red fluorescence emission, indicating the presence of KRT1+ aggressive tumour cells. We also detected that some KRT1+ cells were in proximity to green-fluorescent Iba1 + TAMs, which suggests their close interaction in aggressive carcinomas. Whereas further studies will be necessary to characterise the exact phenotype of TAMs in these tumours, we demonstrate the utility of the peptide **8** in fluorescence imaging studies aimed at understanding dynamic interactions between immune cells and cancer cells in tumour tissues *in situ*.

## Conclusions

In summary, we describe the preparation of Trp(redBODIPY) as the first red-emitting Trp-based amino acid by Pd-catalysed coupling to a thiophene-appended BODIPY fluorophore. Trp(redBODIPY) is compatible with conventional SPPS protocols and it was used to synthesize a minimally-disrupted KRT1-binding peptide with red fluorogenic properties. Notably, our computational and experimental data indicates that the incorporation of Trp(redBODIPY) within KRT1-binding sequences is more favourable than the conventional lysine derivatisation with BODIPY TR, in terms of binding mode, protein affinity and signal-to-noise ratios. Finally, we used the Trp(redBODIPY)-labelled cyclopeptide **8** for live-cell and *ex vivo* imaging of KRT1+ cancer cells and to study their interactions with tumour-associated macrophages in aggressive carcinomas.

## Ethical statement

This study was performed in strict accordance with the guidelines on the United Kingdom Animals Act 1986 for the care and use of laboratory animals. The work was approved by the Home Office (Animals in Science Regulation Unit) and performed under the local rules and guidance of the Animal Welfare and Ethical Review Body at The University of Edinburgh (Edinburgh, UK), which is a designated User and Supplying Establishment under ASPA (1986) with PEL number 60/6205.

## Conflicts of interest

The authors declare no conflicts of interest.

## Supplementary Material

SC-011-C9SC05558D-s001
