# Supplementary material for: Fluorogenic Trp(redBODIPY) cyclopeptide targeting keratin 1 for imaging of aggressive carcinomas
Source: Chem Sci. 2019 Dec 18;11(5):1368–74. doi: 10.1039/c9sc05558d (PMC8148049; doi:10.1039/c9sc05558d)
Supplement: SC-011-C9SC05558D-s001 [file SC-011-C9SC05558D-s001.pdf]

Electronic Supplementary Material (ESI) for Chemical Science.
This journal is © The Royal Society of Chemistry 2019

Electronic Supplementary Information

Fluorogenic Trp(redBODIPY) Cyclopeptide Targeting Keratin 1 for Imaging of Aggressive Carcinomas

Table of Contents

1. Chemical synthesis.
2. NMR and HRMS characterisation.
3. Computational analysis.
4. Supplementary Figures and Tables.
5. Experimental protocols for biological assays.
6. Supplementary references.

1. Chemical synthesis

Reactions were monitored by HPLC-MS at 220 nm using a HPLC Waters Alliance HT comprising a pump (Edwards RV12) with degasser, an autosampler and a diode array detector. Flow from the column was split to a MS spectrometer. The MS detector was configured with an electrospray ionisation source (micromass ZQ4000) and nitrogen was used as the nebuliser gas. Data acquisition was performed with MassLynx software. For new peptides, yields are estimated from the integration of the peak areas in the HPLC-MS crude. Other yields are for the isolated pure compound. All microwave reactions were carried out in 10 mL sealed glass tubes in a focused mono-mode microwave oven ("Discover" by CEM Corporation) featured with a surface sensor for internal temperature determination. Cooling was provided by compressed air ventilating the microwave chamber during the reaction. When stated, the final crude was purified via flash column chromatography Combi Flash ISCO RF provided with dual UV detection.

NMR spectra were acquired with either a Bruker DMX-500 MHz spectrometer MHs. The spectra were referenced relative to the solvent residual signal. ^{1}H resonances were unequivocally assigned by two-dimensional NMR experiments (COSY, TOCSY and NOESY and/or ROESY). Then, the ^{13}C resonances were straightforwardly assigned on the basis of the cross-correlations observed in the ^{1}H-^{13}C HSQC spectra. Chemical shifts (δ) are reported in ppm. Multiplicities are referred by the following abbreviations: s = singlet, d = doublet, t = triplet, dd = double doublet, dt = double triplet, q = quartet, p = pentuplet and m = multiplet. HRMS (ESI positive) were obtained with a LTQ-FT Ultra (Thermo Scientific) mass Spectrometer. Commercially available reactants were used without further purification. Thin-layer chromatography was conducted on Merck silica gel 60 F254 sheets and visualised by UV (254 nm and 365 nm). Silica gel (particle size 35–70 μm) was used for flash column chromatography. Spectral properties (absorption/emission) of amino acid and peptides were recorded using a BioTek Cytation 3 Imaging Reader. UV-Vis absorption measurements were performed using Thermo Scientific Nanodrop 1000 Spectrophotometer. HPLC-MS analysis were performed on a Waters HPLC system comprised of a Waters Alliance 2695 separation module, Waters 2996 photodiode array detector, coupled to a Waters Micromass ZQ Mass Spectrometer with electrospray ionisation source, in conjunction with Masslynx v4.1 programme.

Fmoc-Glu(OBzl)-OH, Fmoc-Tyr(Bzl), Fmoc-Nle-OH, Fmoc-Gln-OH, Fmoc-Lys(Z)-OH were obtained from Sigma-Aldrich. Fmoc-D-Lys(Z)-OH was purchased from Cambridge BioScience. The remaining Fmoc-amino acids were obtained from Iris Biotech. Coupling reagents COMU, PyOxim and Oxyma Pure were kindly provided by Luxembourg

Biotechnologies. Proteolytic assays were performed using a *Streptomyces griseus* protease cocktail (type XIV) from Sigma-Aldrich. 2-chlorotrityl chloride polystyrene resin was obtained from Merck Novabiochem. Completion of peptide coupling was checked using a commercial Ninhydrin kit (AnaSpec) at 120ºC in a heating block inside a fume hood. Kinetex 150 × 4.6 mm^2 (5 µm) C18 column was employed, together with H_2O (0.1% HCOOH) and CH_3CN (0.1% HCOOH) as eluents. Purifications were conducted in a semi-Preparative Waters HPLC consisting of a Waters 2707 autosampler and a Waters 2489 detector (210-600 nm). Kinetex Axia 150 × 21.2 mm^2 (5 µm) C18 column was used, together with H_2O (0.1% HCOOH) and CH_3CN (0.1% HCOOH) as eluents and flow rate 10 mL min^{-1}.

General procedures for SPPS[1]

Peptides were manually synthesized in 10-mL polystyrene syringes fitted with a polyethylene porous disc using common Fmoc-SPPS protocols. Solvents, excess of reagents and soluble byproducts were removed by suction. The Fmoc group was removed with piperidine/DMF (1:4) (1 × 1 min, 2 × 5 min), followed by DMF (x5), and DCM (x5) washes. All syntheses were carried out at room temperature. Peptides bearing fluorescent moieties were always protected from light.

Resin loading: Fmoc-Ala-OH·H_2O (1.2 eq.) was loaded onto 2-chlorotrityl polystyrene resin (1 eq.) using DIPEA (3.6 eq.) in DCM for 10 mins followed by additional DIPEA (8.4 eq.) for extra 40 mins. MeOH (50 µl) was added to cap remaining trityl groups. The resin was then filtered and washed using MeOH/DIEA/DCM 10:5:85 (3 × 1 min), followed by DCM (5 × 1 min) and DMF (5 x 1 min). The loading of the resin was determined by measuring the absorbance of piperidine-dibenzofulvene adduct at 301 nm using Nanodrop, as an indirect reading of the extent of amino acid coupling.

Peptide elongation: After the Fmoc group was removed, the resin was washed with DMF (4 × 1 min), DCM (3 × 1 min), DMF (4 × 1 min). Unless otherwise stated, coupling was carried out using Fmoc-AA-OH (4 eq.), COMU (4 eq.), Oxyma (4 eq.) and DIPEA (8 eq.) in DCM/DMF (1:1) for 1 h with 5 min pre-activation. The resin was then washed with DMF (5 × 1 min), DCM (5 × 1 min) and filtered. The completion of the coupling step was confirmed using Kaiser Test. The resin was then washed with DMF (5 × 1 min) followed by treatment with Ac_2O/DIPEA/DMF 5:6:89 (3 × 2 min) to cap the free N-terminus, and washes with DMF (5 × 1 min), DCM (5 × 1 min), DMF (5 × 1 min). Before the next coupling cycle, Fmoc group is removed as described above.

Cleavage from resin: The peptide was then cleaved from the resin using 2% TFA in DCM (5 × 1 min) and washed with DCM (5 × 1 min). The combined filtrates were collected into a 150-ml

round bottom flask containing DCM (50 mL) and DIEA (250 µL). Solvent was evaporated under reduced pressure and the remaining residue was dissolved in CH_3CN/H_2O 1:1 and lyophilised.

Cyclisation: Cleaved peptide (1 eq.) was dissolved in DCM/DMF (1:4, 0.055 M), followed by stepwise addition of PyOxim (1.4 eq.) and Oxyma (1.2 eq.). After setting the cocktail at -10 ˚C using a salted ice bath, DIEA (2.5 eq.) was added and the mixture stirred for 2-4 h at -10 ˚C. Excess PyOxim was removed from the cyclised product by precipitation using cold water. The precipitate was washed with H_2O, decanted and dried.

tert-Butyl 2-(5-methylthiophen-2-yl)-1H-pyrrole-1-carboxylate (5)

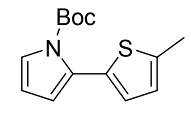

5

This product was prepared following a described procedure for 2-arylation of pyrroles.[2] To a stirred solution of 2-bromo-4-methyltiophene (0,637 g), N-(Boc) pyrrole-2-boronic acid (1.06 g, 1.2 eq.) and tetrakis-(triphenylphosphine)palladium (0.21 g, 0,05 eq) in 1,2-dimethoxyethane (100 mL) at room temperature under nitrogen was added a solution of sodium carbonate (1.6 g, 4,2 eq.) in water (8 mL). The mixture was heated at reflux for 4 h, cooled, then partitioned between water (300 mL) and dichloromethane (4 × 100 mL). The combined extracts were dried (Na_2SO_4) and evaporated in vacuo to give an oil. Chromatography in flash silica with gradient elution using 10 - 50% dichloromethane in hexane gave the desired product **5** (0.93 g, 98%) as a colourless solid. ^{1}H NMR (400 MHz, $CDCl_3$) δ = 7.33 (dd, *J* = 3.4, 1.8 Hz, 1H), 6.84 (d, *J* = 3.4 Hz, 1H), 6.66 (m, *J* = 3.3, 1.0 Hz, 1H), 6.26 (dd, *J* = 3.3, 1.8 Hz, 1H), 6.19 (t, *J* = 3.4 Hz, 1H), 2.48 (d, *J* = 1.0 Hz, 3H), 1.45 (s, 9H). ^{13}C NMR (101 MHz, $CDCl_3$) δ = 149.0, 140.1, 132.4, 127.6, 127.5, 124.6, 122.8, 116.1, 110.4, 83.6, 27.7, 15.3. HRMS: calculated for $C_{14}H_{18}NO_2S^+$ [M+H]$^+$ 264.1053; found: 264.1055.

2-(5-Methylthiophen-2-yl)-1H-pyrrole (4)

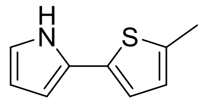

4

The deprotection of the Boc protecting group was performed following the described methodology.[3] A dry, 50-mL, round-bottomed flask equipped with magnetic stirrer was maintained under a nitrogen atmosphere. The protected pyrrole **5** (1 g) was added followed by THF (18 mL) . The mixture was stirred at room temperature and 3 equiv. of $NaOCH_3$, in CH_3OH (6.5 N) were added. The mixture was allowed to stir 1 h and was then diluted with 5 mL of Et_2O and 5 mL of H_2O. Separation of the organic layer followed by washing with brine, drying over $MgSO_4$, and rotary evaporation gave pure compound **4** as a dark grey powder in quantitative yield. ^{1}H NMR (400 MHz, $CDCl_3$) δ = 8.23 (bs, 1H), 6.81 (d, *J* = 3.5 Hz, 1H), 6.78 (m, *J* = 2.7, 1.5 Hz, 1H), 6.66 (m, *J* = 3.4, 1.1 Hz, 1H), 6.34 (m, *J* ≈ 3.5 Hz, 1H), 6.25 (m, *J* = 6.1, 2.7 Hz, 1H), 2.48 (d, *J* = 1.1 Hz, 3H). ^{13}C NMR (101 MHz, $CDCl_3$) δ = 137.4, 133.9, 125.6, 120.7, 118.1, 109.8, 106.1, 15.2. HRMS: calculated for $C_9H_{10}NS^+$ [M+H]$^+$ 164.0528; found: 164.0538.

(3,5-Dimethyl-1H-pyrrol-2-yl)(3-iodophenyl)methanone (6)

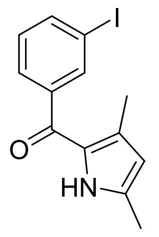

6

This compound was prepared using the described methodology for the preparation of acid chlorides.[4] 3-iodobenzoic acid (1 g, 4 mmol) was dissolved in anhydrous CH_2Cl_2 (40 mL) and cooled with ice water. Oxalyl chloride (0,51 mL, 1.5 eq.) was added dropwise to the solution followed with the addition of catalytic amount of DMF (4 drops). The resulting mixture was allowed to stir at room temperature for additional 2 h and the solvent was evaporated to afford crude acid acid chloride, which was dissolved CH_2Cl_2 anhydrous (30 mL). 2,4-dimethylpyrrole (0,42 mL, 1 eq.) was added dropwise during 5 min. The mixture was stirred at room temperature for 3 hours and partitioned in saturated $NaHCO_3$ aqueous solution (15 mL) and CH_2Cl_2 (25 mL), the organic extracts were dryed over $MgSO_4$ and the solvent evaporated. The residue was purified by flash column cromatography with 40-90% CH_2Cl_2 in hexane to afford the desired ketone **6** as a pale yellow powder (362 mg, 27%). ^1H NMR (400 MHz, $CDCl_3$) δ = 8.98 (bs, 1H), 7.95 (t, J = 1.6 Hz, 1H), 7.83 (m, J = 7.8, 1.8, 1.1 Hz, 1H), 7.58 (m, J ≈ 7.8 Hz, 1H), 7.19 (t, J = 7.8 Hz, 1H), 5.88 (d, J = 2.9 Hz, 1H), 2.30 (s, 3H), 1.93 (s, 3H). ^{13}C NMR (101 MHz, $CDCl_3$) δ = 183.9, 142.3, 139.9, 137.4, 136.6, 131.3, 130.3, 127.7, 127.5, 113.6, 94.2, 14.4, 13.5. HRMS: calculated for $C_{13}H_{13}INO^+$ $[M+H]^+$ 326.0036; found: 326.0040.

5,5-Difluoro-10-(3-iodophenyl)-1,3-dimethyl-7-(5-methylthiophen-2-yl)-5H-dipyrrolo[1,2-c:2',1'-f][1,3,2]diazaborinin-4-ium-5-uide (3)

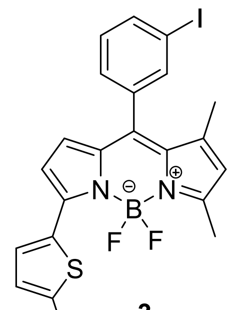

3

The iodinated BODIPY derivative 3 was obtained by adaptation of a reported syntethic procedure.[5] Ketone **6** (670 mg, 5 mmol) was dissolved in CH_2Cl_2 (10 mL) and stirred under nitrogen. Then pyrrole **5** (620 mg, 5 mmol, 1,2 equiv.) was added and the resulting solution was cooled to 0 °C in an ice bath, followed by the addition of $POCl_3$ (0,13 mL, 1,3 equiv.). The solution was stirred at room temperature overnight. Triethylamine (1,4 mL, 10 eq.) was added and the mixture was stirred for 10 min. while being cooled to 0 °C. Boron trifluoride–diethyl ether (1,5 mL, 12 eq.) was added dropwise and the reaction mixture was than stirred at room temperature overnight. The dark violet solution was poured into aqueous $NaHCO_3$ saturated solution (20 mL). and extracted with CH_2Cl_2 (3 × 15 mL). The organic layer was dried with $MgSO_4$, filtered, and concentrated in vacuo. The crude residue was purified by flash column chromatography on silica, 0-40% CH_2Cl_2 in hexane to yield compound **3** as a black powder (122 mg, 22%). ^1H NMR (400 MHz, $CDCl_3$) δ = 7.88 (d, *J* = 3.8 Hz, 1H), 7.84 (m, *J* = 7.8, 1.5, 1.0 Hz, 1H), 7.73 (t, *J* = 1.5 Hz, 1H), 7.33 (d, *J* = 7.8 Hz, 1H), 7.22 (t, *J* = 7.8 Hz, 1H), 6.83 (dd, *J* = 3.8, 1.0 Hz, 1H), 6.61 (d, *J* = 4.3 Hz, 1H), 6.40 (d,

J = 4.3 Hz, 1H), 6.10 (s, 1H), 2.63 (s, 3H), 2.53 (s, 3H), 1.55 (s, 3H). ^{13}C NMR (101 MHz, $CDCl_3$) δ = 159.1, 148.7, 143.9, 143.6, 138.2, 138.2, 137.7, 136.4, 132.0, 130.7, 130.6, 130.6, 130.0, 128.7, 128.3, 127.4, 122.6, 118.6, 93.8, 15.5, 15.1, 14.9. ^{19}F NMR (376 MHz, $CDCl_3$) δ = -143.1 (m). HRMS calculated for $C_{22}H_{19}BF_2IN_2S^+$ $[M+H]^+$ 519.0369; found: 519.0384.

[5,5'-bis(5-methylthiophen-2-yl)-3',4'-dihydro-1H,2'H-2,3'-bipyrrole (7)

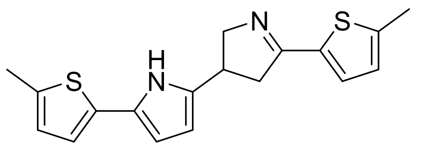

7

In this latter process, the dimerisation of the pyrrole-tiophene precursor was observed. During chromathography the byproduct **7** [5,5'-bis(5-methylthiophen-2-yl)-3',4'-dihydro-1H,2'H-2,3'-bipyrrole] was isolated as a brown powder in a 50% yield. ^{1}H NMR (400 MHz, $CDCl_3$) δ = 8.24 (bs, 1H), 7.09 (d, J = 3.6 Hz, 1H), 6.77 (d, J = 3.5 Hz, 1H), 6.71 (dd, J = 3.6, 1.0 Hz, 1H), 6.62 (dd, J = 3.5, 1.1 Hz, 1H), 6.20 (t, J = 3.0 Hz ,1H), 5.97 (t, J = 3.0 Hz, 1H), 4.38 (dd, J = 16.1, 8.5 Hz, 1H), 4.07 (dd, J = 16.1, 6.1 Hz, 1H), 3.68 (m, 1H), 3.34 (dd, J = 16.8, 9.8 Hz, 1H), 3.05 (m, J = 16.8, 6.8, 1.8 Hz, 1H), 2.51 (s, 3H), 2.45 (d, J = 1.0 Hz, 3H). ^{13}C NMR (101 MHz, $CDCl_3$) δ = 167.31, 144.91, 137.18, 136.85, 134.89, 134.12, 130.05, 126.63, 126.06, 125.73, 120.47, 106.30, 106.22, 67.38, 43.11, 36.58, 15.91, 15.39. HRMS calculated for: $C_{18}H_{19}N_2S_2^+$ $[M+1]^+$ 327.0984; found: 327.0996.

10-(3-(3-(2-((((9H-Fluoren-9-yl)methoxy)carbonyl)amino)-2-carboxyethyl)-1H-indol-2-yl)phenyl)-5,5-difluoro-1,3-dimethyl-7-(5-methylthiophen-2-yl)-5H-dipyrrolo[1,2-c:2',1'-f][1,3,2]diazaborinin-4-ium-5-uide (1)

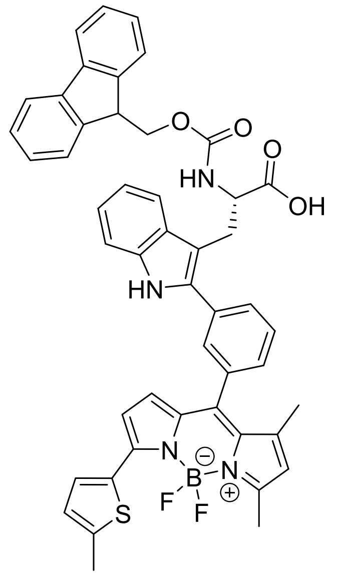

1

Fmoc-L-Trp-OH (69 mg, 0,16 mmol), BODIPY **3** (100 mg, 1,2 eq.), $AgBF_4$ (31 mg, 1 eq.) and $Pd(OAc)_2$ (7,2 mg, 0.2 eq.) were dissolved in DMF (2 mL). and stirred for 5 minutes. 1 eq. of trifluoroacetic acid was added to the mixture, wich was allowed to react in a microwave reactor for 30 minutes at 80 ºC. After this time, $AgBF_4$ (1 eq.) and $Pd(OAc)_2$ (0,2 eq.) were added and the mixture was set to react another cycle of 30 min at 80 ºC.[6] The solvent was evaporated under reduced pressure, the residue dissolved in CH_2Cl_2 (40 mL) and poured on aqueous saturated NH_4Cl solution (30 mL). The organic phase was extracted with CH_2Cl_2 (3 × 25 mL), dryed over $MgSO_4$ and evaporated under reduced pressure. The crude residue was purified by flash column chromatography on silica, 0-3% MeOH in CH_2Cl_2 to yield the compound **1** as a black powder (63 mg, 48%). ^{1}H NMR (400 MHz, $CDCl_3$,data for the major rotamer) δ = 8.47 (d, J = 4.8 Hz, 1H), 7.86 (d, J = 3.7 Hz, 1H), 7.76 – 7.64

(bm, 4H), 7.53 (s, 1H), 7.45 (t, J = 7.7 Hz, 3H), 7.40 – 7.31 (m, 3H), 7.30 – 7.16 (m, 4H), 7.11 (t, J = 7.5 Hz, 1H), 6.80 (d, J = 3.6 Hz, 1H), 6.57 – 6.47 (m, 2H), 6.03 (d, J = 10.1 Hz, 1H), 5.29 (t, J = 7.4 Hz, 1H), 4.61 (m, 1H), 4.23 (m, 1H), 4.16 (m, 1H), 4.06 (m, 1H), 3.50 (m, 2H), 2.61 (s, 3H), 2.50 (s, 3H), 1.54 (s, 3H). ^{19}F NMR (376 MHz, DMSO) δ = -140.72 (m). HRMS calculated for $C_{48}H_{39}BFN_4O_4S^+$ [M-F]$^+$ 797.2764; found: 797.2774.

Unlabelled peptide (2)

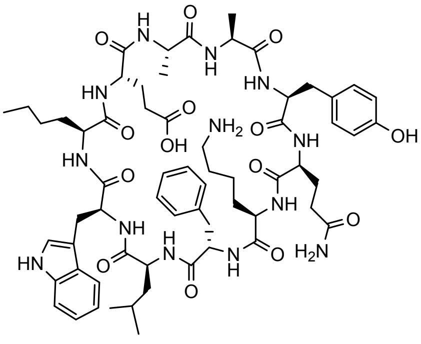

2

The synthesis was performed on 87 mg of 2-chlorotrityl polystyrene resin (0.65 mmol/g), with an initial Ala loading of 56.6 µmol. Fmoc-Tyr(tBu)-OH, Fmoc-Gln(Trt)-OH, Fmoc-D-Lys(Boc)-OH, Fmoc-Trp(Boc)-OH and Fmoc-Glu(OtBu)-OH were used as side-chain protected building blocks. After cleavage and cyclisation as described above, the protected cyclic peptide was transferred onto a 150 ml round bottom flask using DMF (2 × 100 µL) and TFA:TIS:H_2O (95:2.5:2.5, 10mL) was added dropwise to avoid excessive fuming. The solution was stirred for 1 h at 0 ˚C. Upon completion of the reaction, DCM (20 mL) was added to the solution and solvent was evaporated under reduced pressure. This process was repeated three times. Purification was carried out by Semi-Preparative HPLC using a 20-60% gradient over 25 min, with detection at 280 nm. Pure fractions were collected and lyophilised to afford pure peptide **2** as an off-white solid (14.1 mg, 11.2 µmol).

Characterisation data. HPLC-MS: t_R=5.43 min (98% purity). HRMS (ESI-) (m/z): $C_{63}H_{86}N_{13}O_{14}$ $[M-H]^-$ 1248.6423; found, 1248.6366. MALDI (m/z): $[M+H]^+$=1250.4, $[M+Na]^+$=1272.3, $[M+K]^+$=1289.3.

Trp(redBODIPY)-labelled peptide (8)

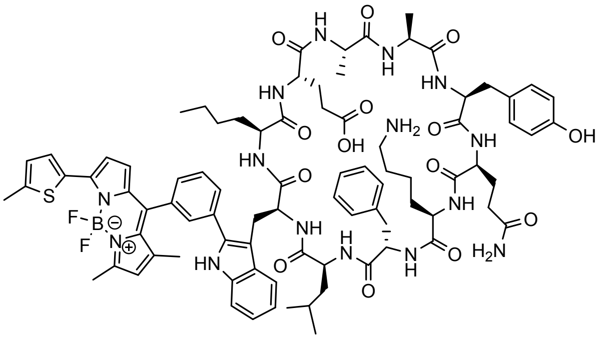

8

Trp(redBODIPY)-labelled peptide was synthesized on 62.5 mg of 2-chlorotrityl polystyrene resin (0.90 mmol/g), with an initial Ala loading of 58.2 µmol. Fmoc-Tyr(Bzl)-OH, Fmoc-D-Lys(Z)-OH, and Fmoc-Glu(OBzl)-OH were used as side-chain protected building blocks. Amino acid **2** (1.5 eq.) was incorporated with COMU (1.5 eq.), Oxyma (1.5 eq.) and DIPEA

(2.0 eq.) in DMF for 2 h. After cleavage from the resin and lyophilisation, the crude linear peptide was purified by semi-Preparative HPLC using a 30-100% gradient over 25 min, with detection at 560 nm. Pure fractions were combined and lyophilised to yield a purple solid (7.2 mg), which was used in the next cyclisation step, as described above. In a similar fashion, the crude cyclic peptide was purified before performing deprotection using a 70-100% gradient over 25 min, with detection at 560 nm. Pure fractions were combined and lyophilised to yield a purple solid (3.1 mg). The crude macrocycle (1 eq, 3.1 mg, 1.6 µmol) was deprotected by means of hydrogenation. Peptide was dissolved in 5% HCOOH/MeOH (1 mlL, and transferred to a 15-mL round bottom flask, followed by addition of 20% $Pd(OH)_2/C$ (1.5 mg, ca. half of peptide mass). Then, the reaction flask was flushed with N_2/vacuum cycles (× 3) and filled with H_2 (1 bar). The reaction mixture was stirred under H_2 at r.t. for 2 h, and upon completion (as checked by HPLC-MS), the catalyst was removed by filtration under Celite and washed with MeOH. Filtrate was collected in a round bottom flask and solvent was removed under reduced pressure. The residue was dissolved in CH_3CN:H2O and lyophilised. Purification was conducted by semi-Preparative HPLC using a 50-80% gradient over 25 min, with detection at 560 nm. Pure fractions were collected and lyophilised to afford pure peptide **8** as a purple solid (0.8 mg, 0.5 µmol).

Characterisation data. HPLC-MS: t_R=6.95 min (99% purity). HRMS (ESI+) (m/z) $C_{85}H_{105}BF_2N_{15}O_{14}S$ $[M+H]^+$ 1640.7742; found: 1640.7432. MALDI (m/z): $[M+Na]^+$=1662.4, $[M+K]^+$=1678.4.

BODIPY TR-labelled peptide (9)

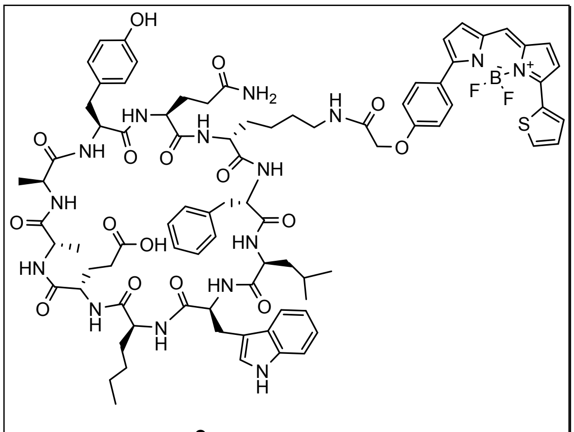

9

Unlabelled peptide **2** (1.1 eq., 7.3 mg, 5.84 µmol) was dissolved in DMF (50 µL). A solution of BODIPY-TR NHS ester (1 eq., 5.35 µmol, 3.4 mg) in DMF (75 µL) was added to the peptide followed by the addition of DIEA (0.5 eq., 2.87 µmol, 0.5 µL). The solution was stirred at room temperature for 1 hour. The crude was directly purified using semi-preparative HPLC using a 50-65% gradient over 25 min, with detection at 560 nm. Pure fractions were collected and lyophilised to afford the pure peptide **9** as a blue solid (8 mg, 4.52 µmol).

Characterisation data. HPLC-MS: t_R=7.63 min (99% purity). HRMS (ESI+) (m/z) $C_{90}H_{112}BF_2N_{16}O_{17}S$ $[M+H]^+$ 1769.8168; found: 1769.7792. MALDI (m/z): $[M+Na]^+$=1792.2, $[M+K]^+$=1807.1.

2. NMR and HRMS characterisation

tert-Butyl 2-(5-methylthiophen-2-yl)-1H-pyrrole-1-carboxylate (5)

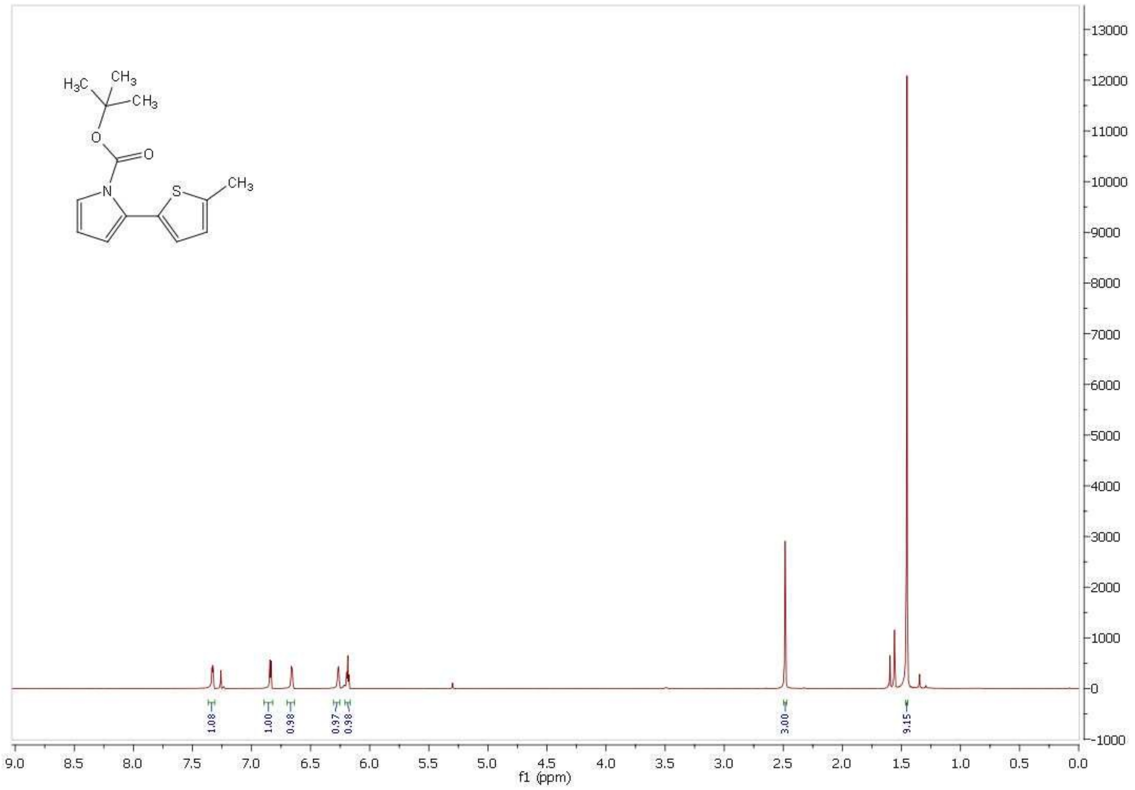

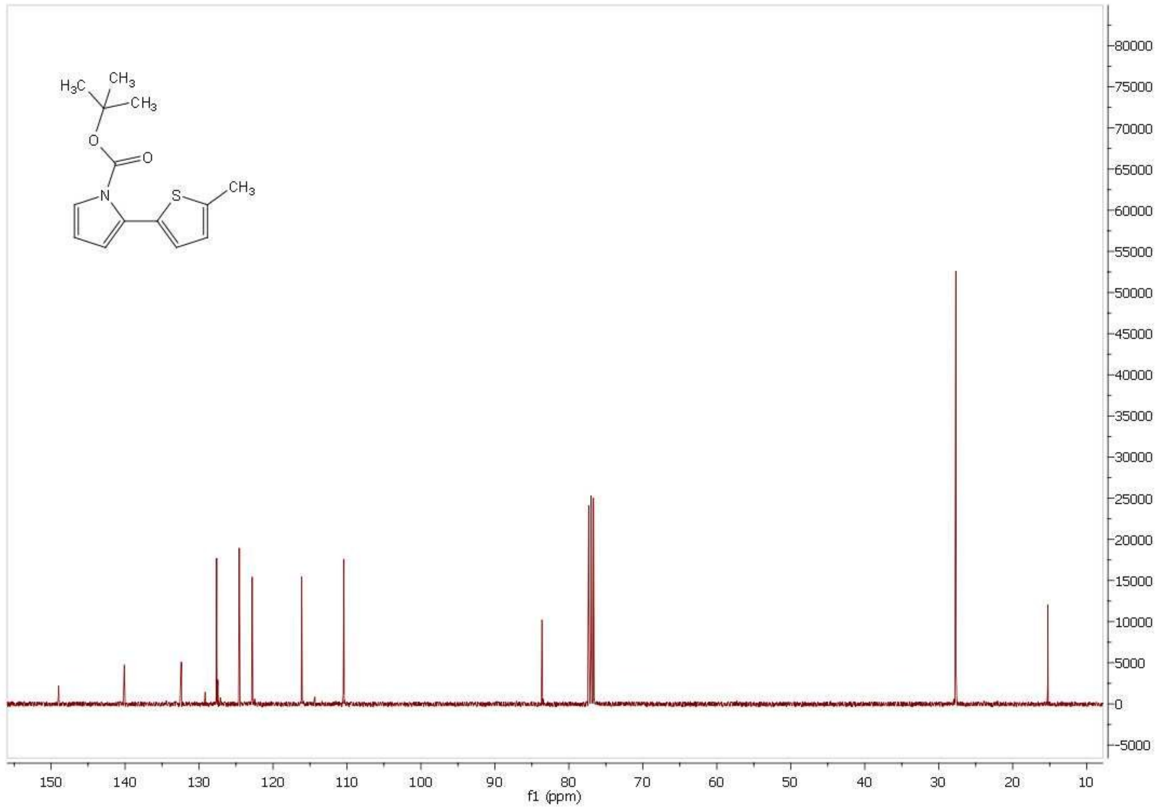

2-(5-Methylthiophen-2-yl)-1H-pyrrole (4)

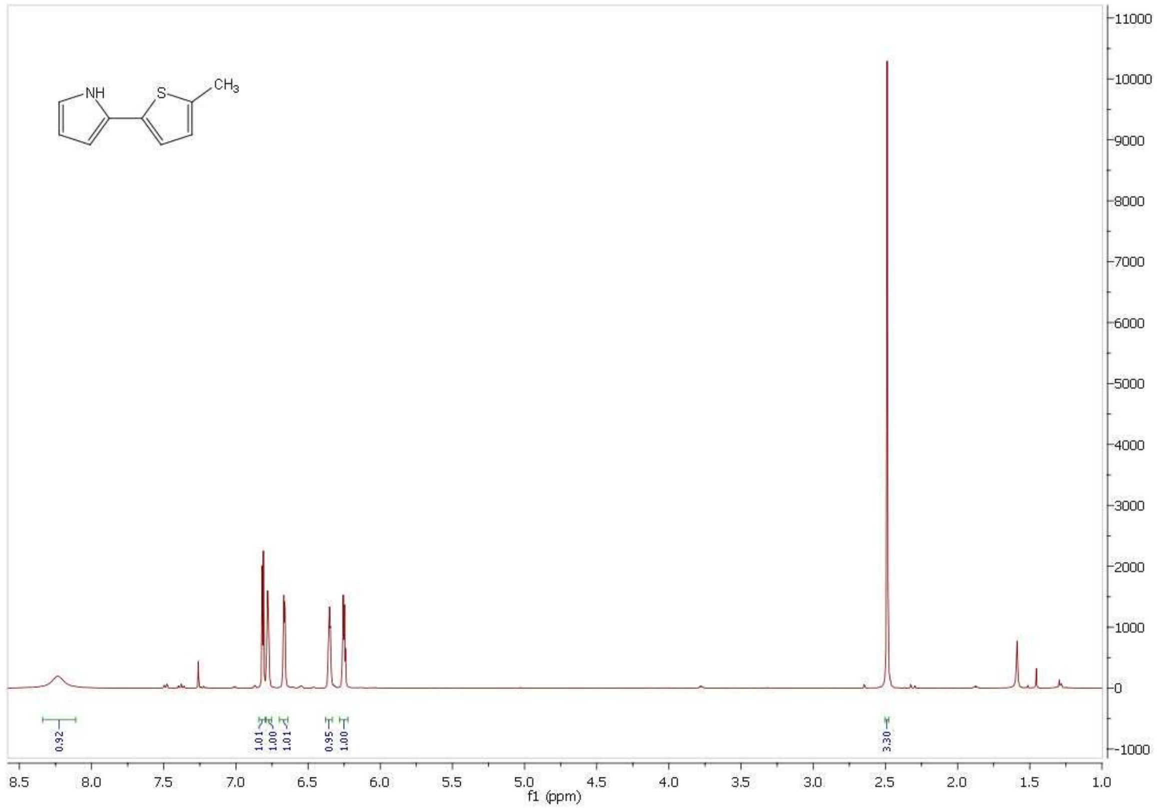

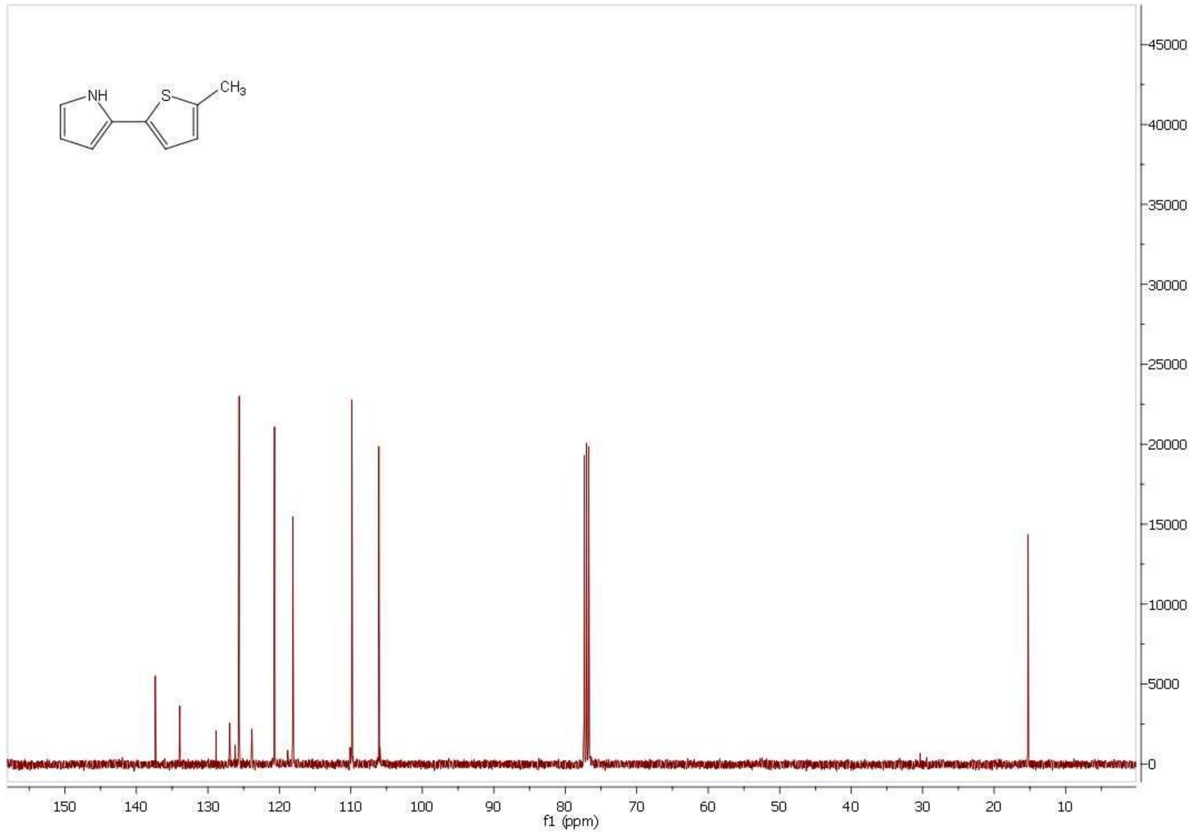

(3,5-Dimethyl-1H-pyrrol-2-yl)(3-iodophenyl)methanone (6)

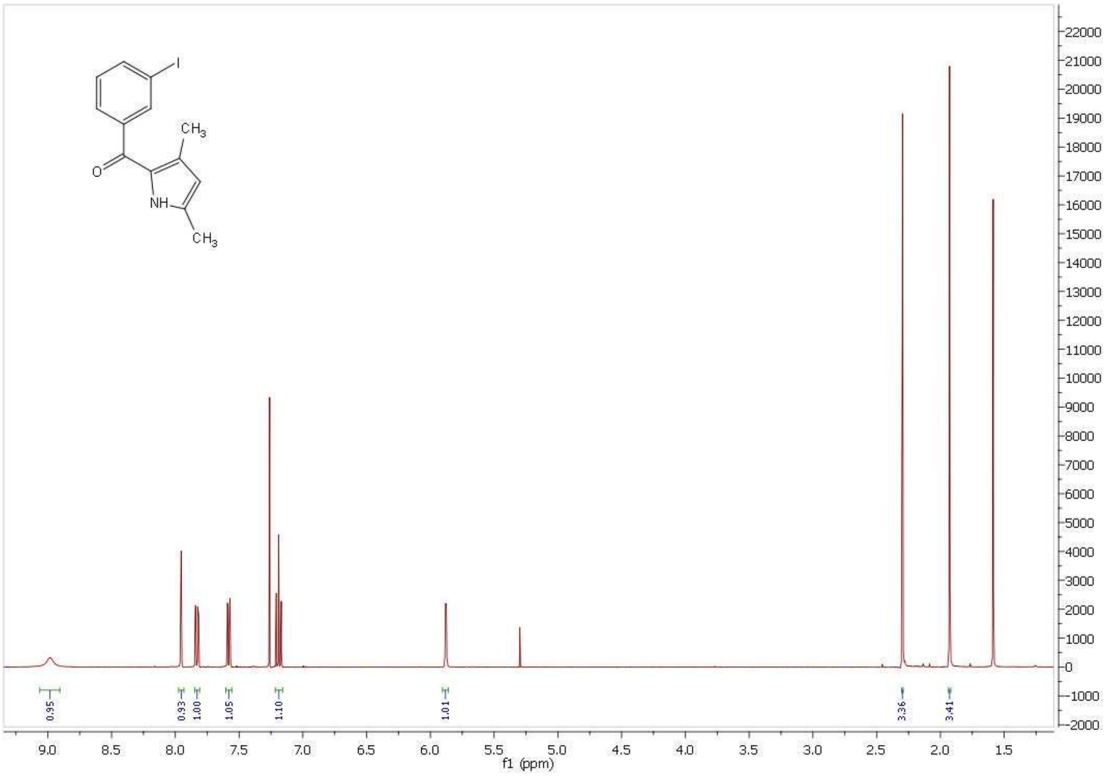

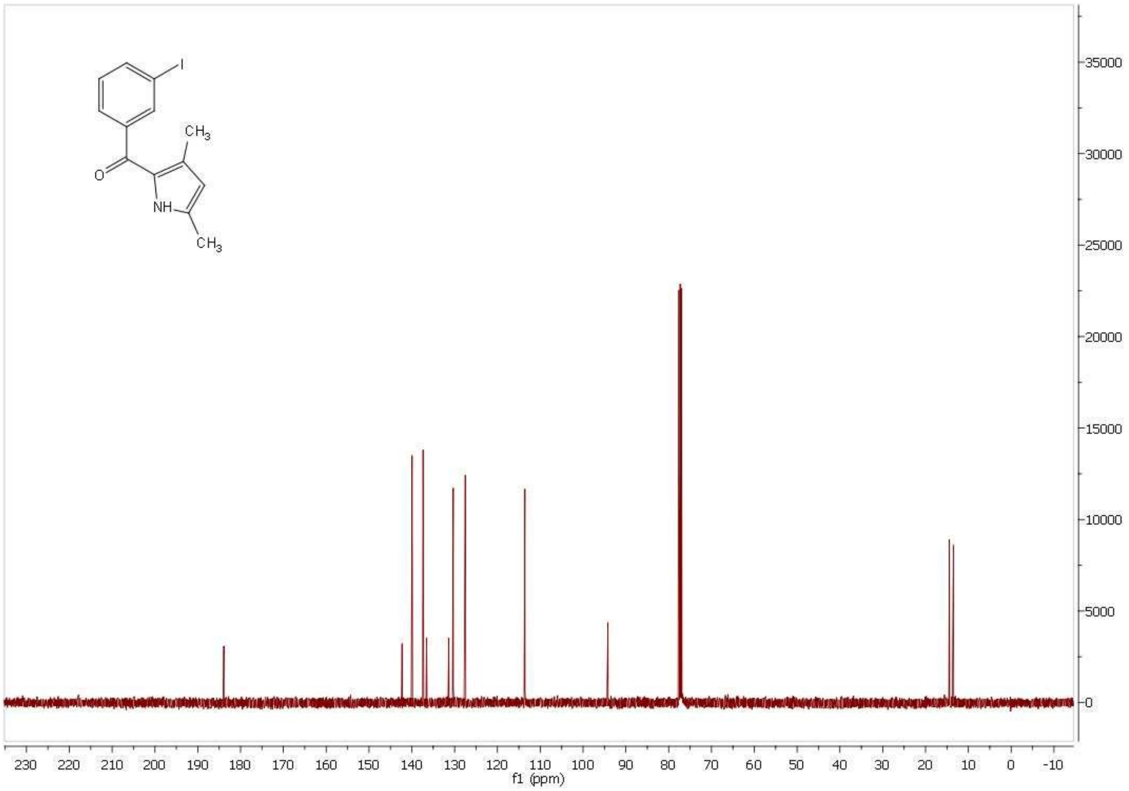

5,5-Difluoro-10-(3-iodophenyl)-1,3-dimethyl-7-(5-methylthiophen-2-yl)-5H-dipyrrolo[1,2-c:2',1'-f][1,3,2]diazaborinin-4-ium-5-uide (3)

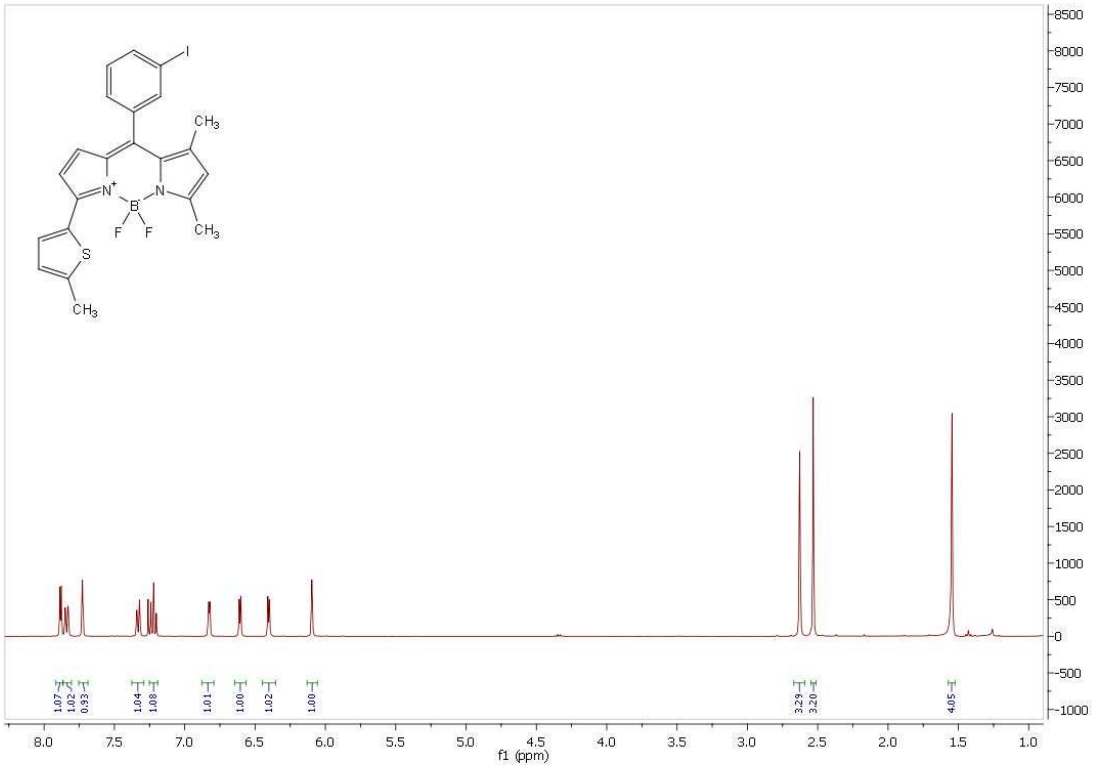

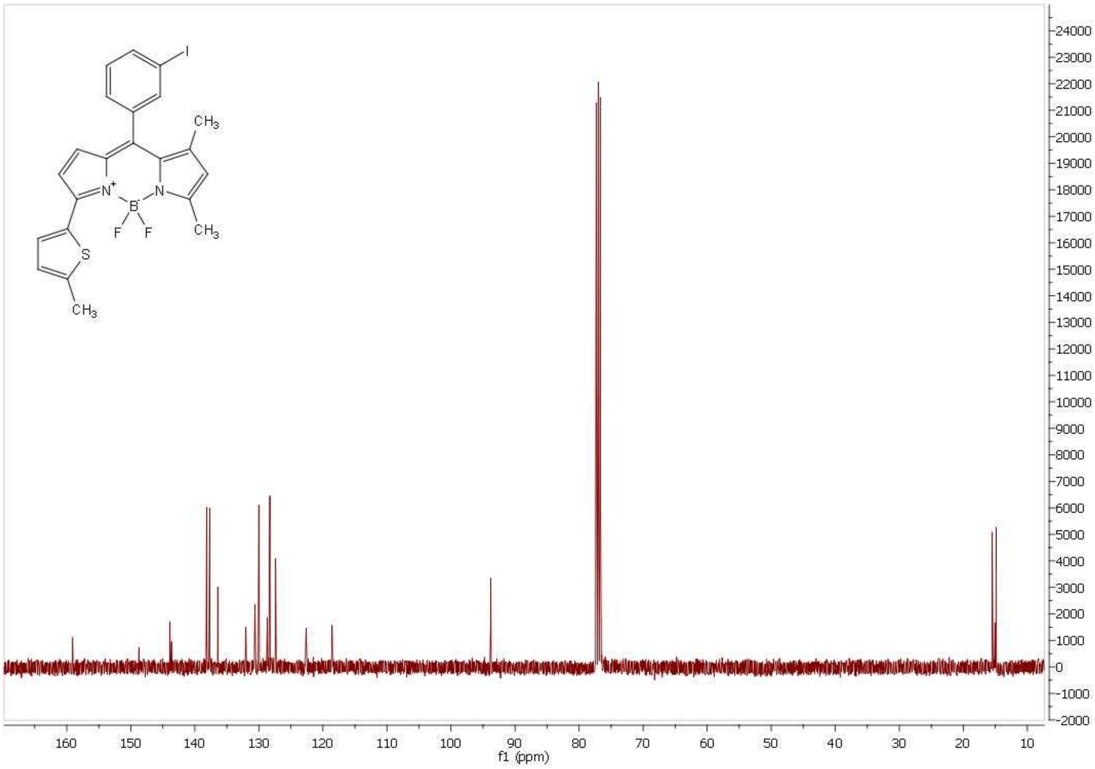

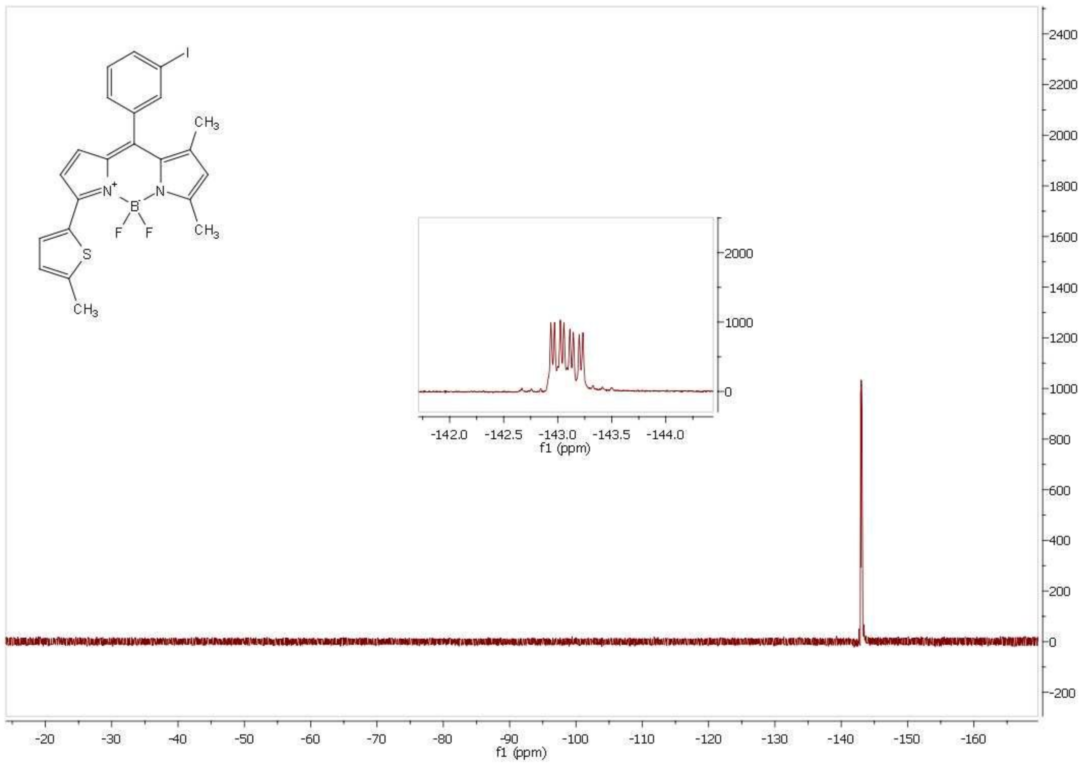

CH3
CH3
CH3
I
N+
B-
N
F
F
S
-142.0
-142.5
-143.0
-143.5
-144.0
f1 (ppm)
2000
1000
0
2400
2200
1800
1600
1400
1200
800
600
400
200
-200
-20
-30
-40
-50
-60
-70
-80
-90
-100
-110
-120
-130
-140
-150
-160

5,5'-bis(5-methylthiophen-2-yl)-3',4'-dihydro-1H,2'H-2,3'-bipyrrole (7)

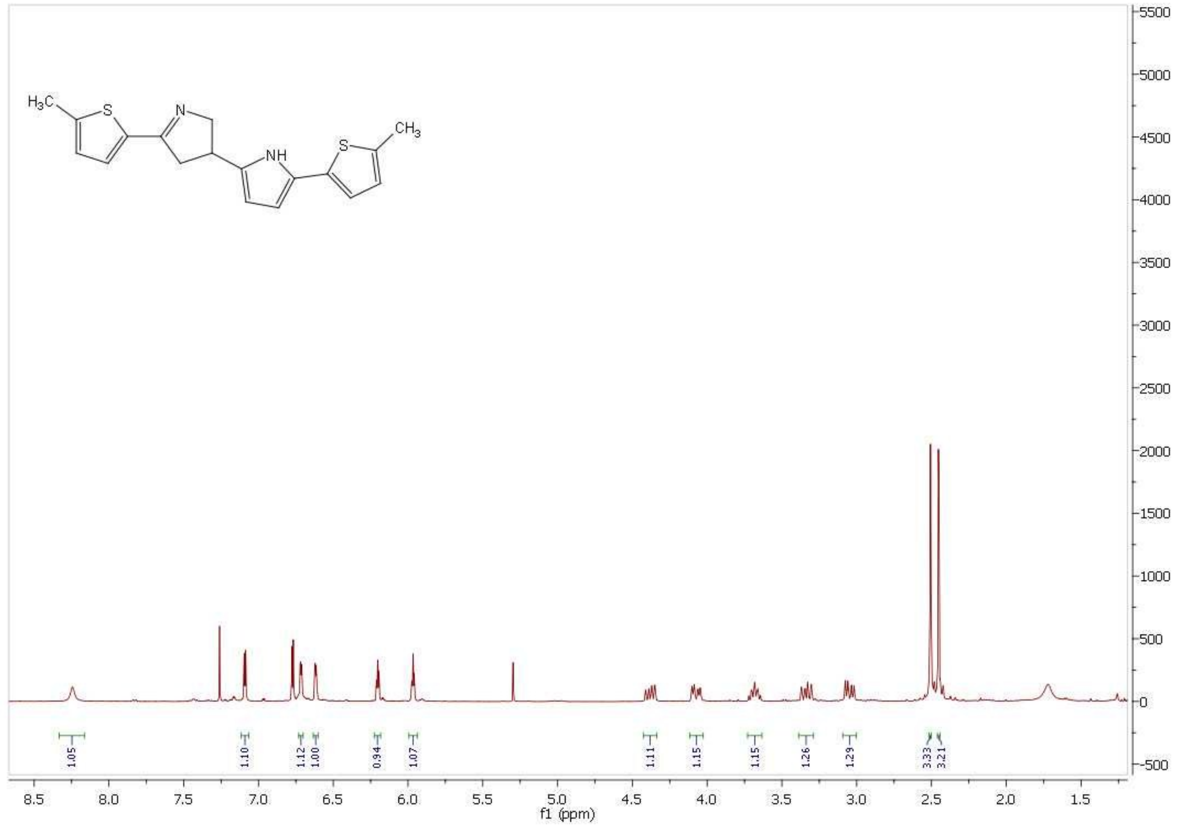

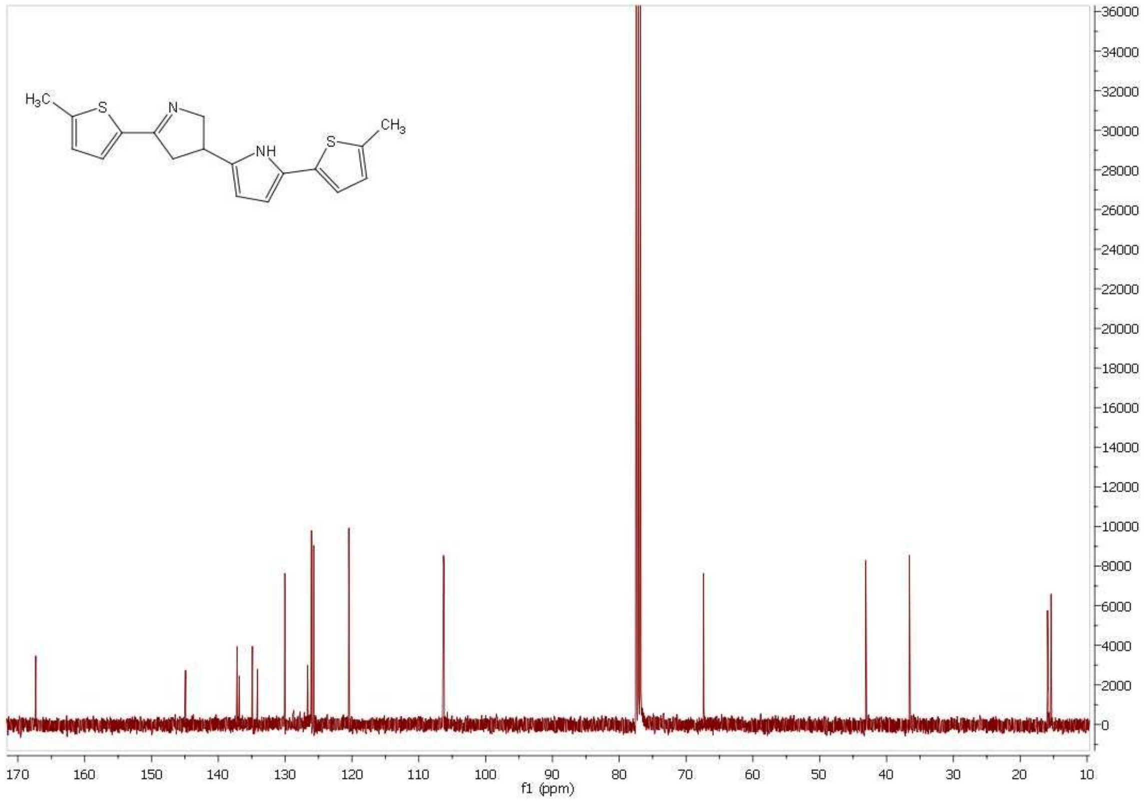

^{1}H gCOSY and HSQC

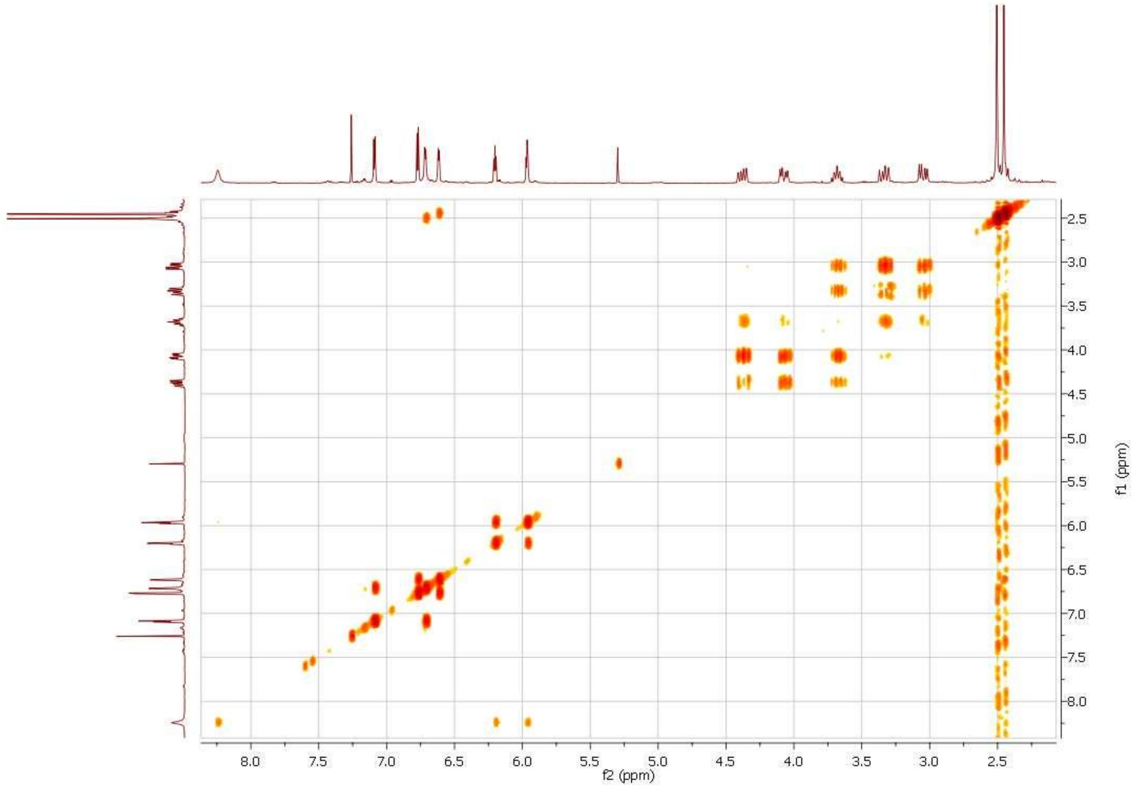

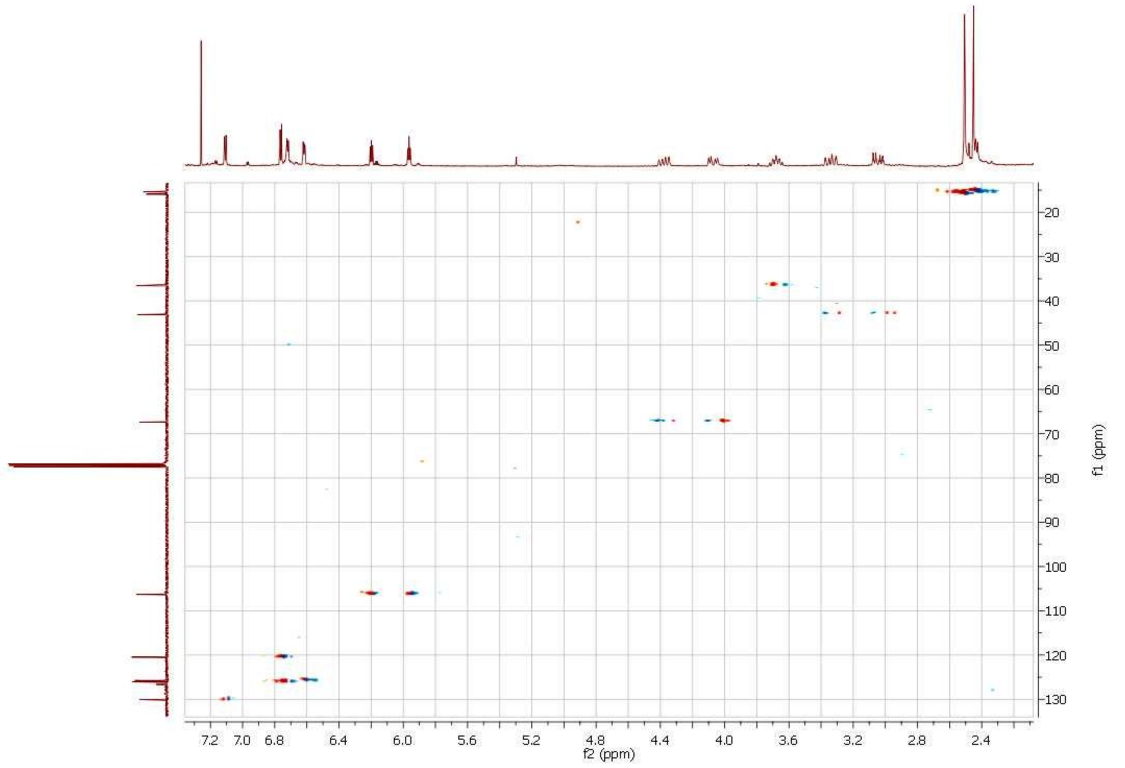

10-(3-(3-(2-((((9H-Fluoren-9-yl)methoxy)carbonyl)amino)-2-carboxyethyl)-1H-indol-2-yl)phenyl)-5,5-difluoro-1,3-dimethyl-7-(5-methylthiophen-2-yl)-5H-dipyrrolo[1,2-c:2',1'-f][1,3,2]diazaborinin-4-ium-5-uide (1) (upper spectrum in $CDCl_3$ containing traces of DMF; bottom spectrum in DMSO).

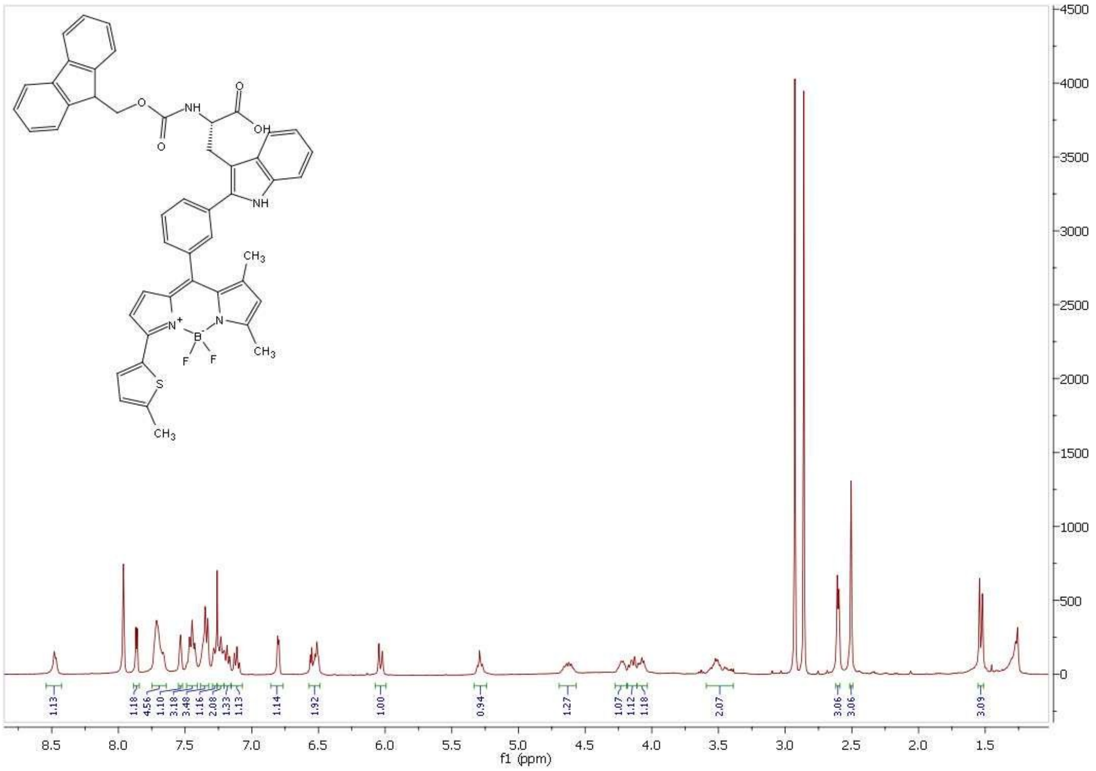

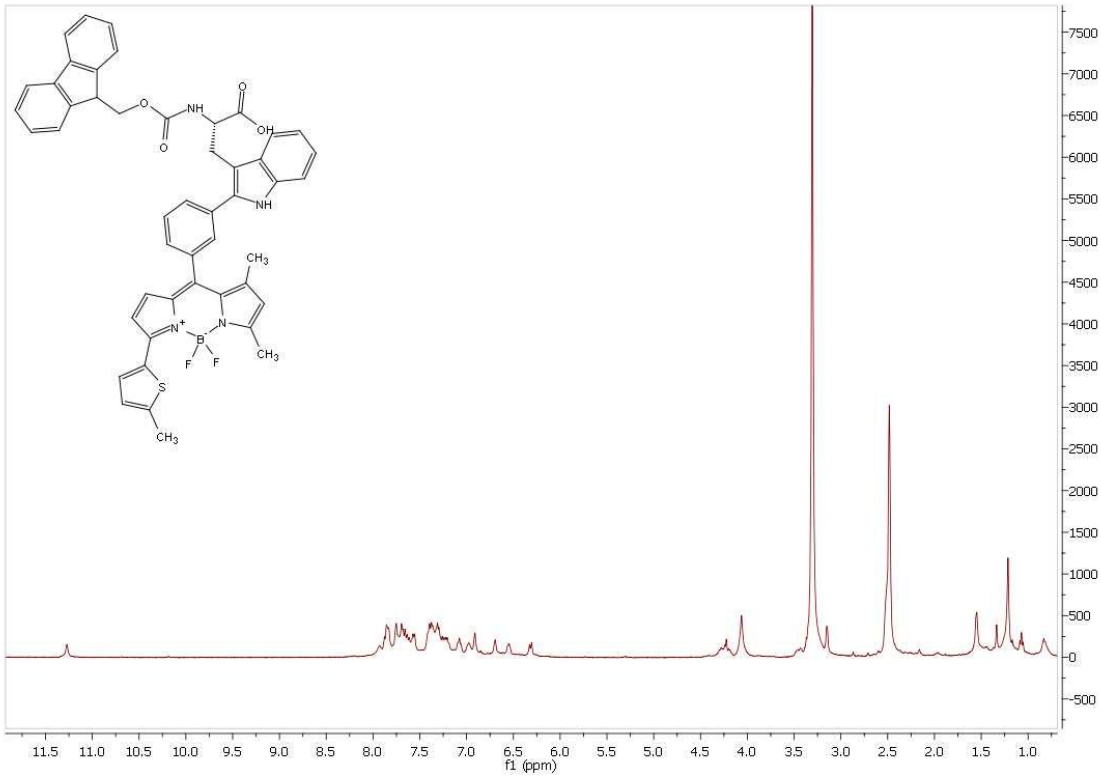

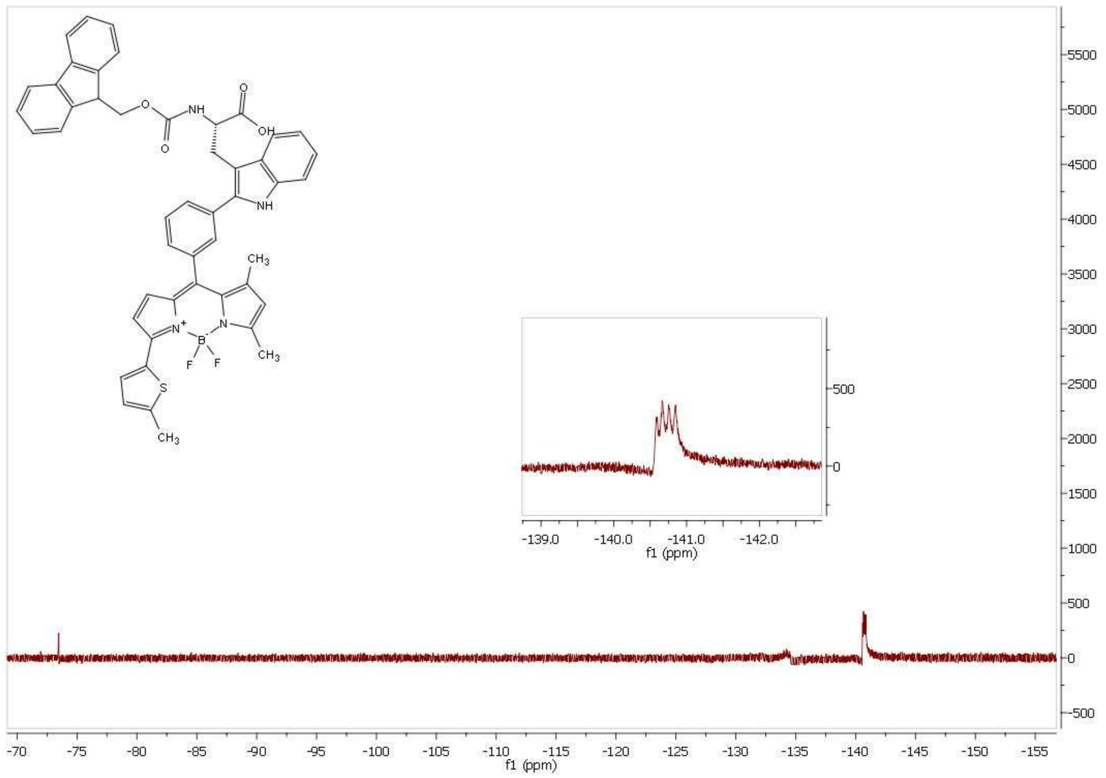

f1 (ppm)

HRMS spectra for peptides 2, 8 and 9

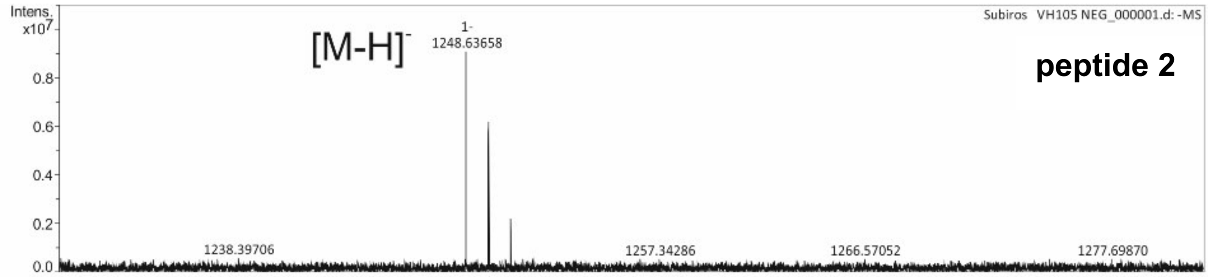

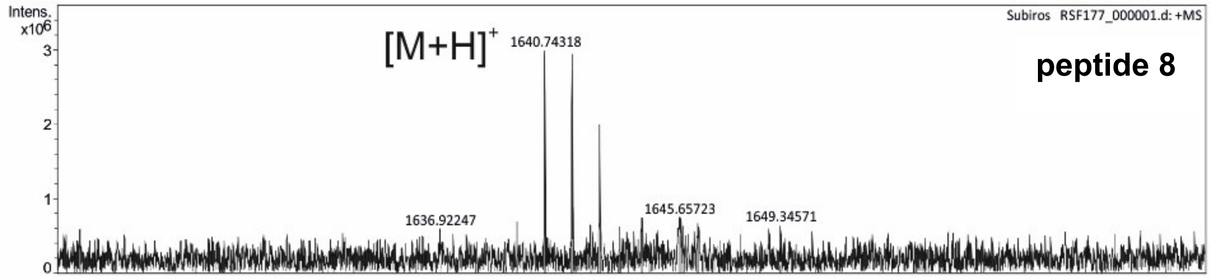

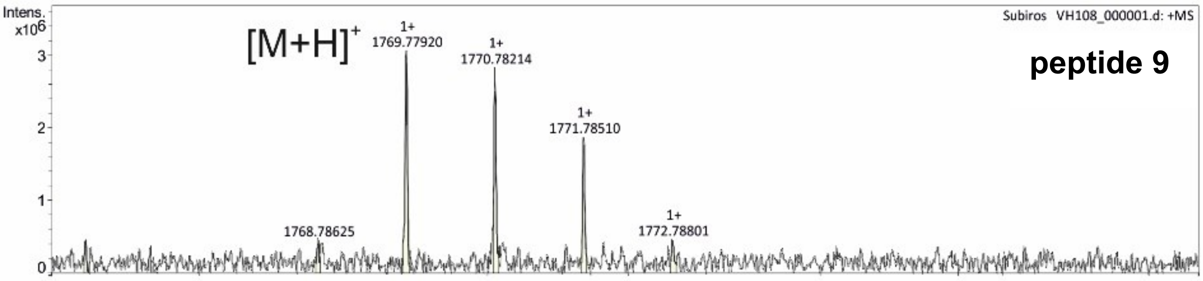

3. Computational Analysis

Identification of the putative binding site

The protein KRT1 has 644 amino acids but there is no available crystal structure for the whole protein. However, two sections (namely coil 1B and coil 2B) have been solved by X-ray crystallography in a heterodimeric form with protein KRT10.[7,8] Biophysical binding experiments (Surface Plasmon Resonance, SPR) have narrowed down the binding of peptide **2** to the coil 2B region of KRT1 (residues 387-496).[9] Thus, we have focussed our analysis on this part of the protein. Firstly, 200ns Molecular Dynamics (MD) simulations in water were performed for the available crystal structure of KRT1 (PDB code:4ZRY[7], chain B; residues 386 to 489). Atomic fluctuation analysis showed very high values (Figure S7), meaning that the protein is moving considerably during the simulation. This was largely predictable, since this part of the protein is a 104 amino acid elongated alpha helix structure. Analysis of the secondary structure through the DSSP [10] software revealed that the protein unfolds completely during the simulation, except for two small regions that preserve their helical structure: residues 472-482 and 456-461 (Figure S8A). On the other hand, we also analysed the sequence conservation of KRT1 (UniProt code P04264), residues 387-496 (coil 2B domain). We performed Multiple Sequence Analysis (MSA) across species. We used ClustalΩ[11] with default settings. Interestingly, the same regions that stayed folded during the MD were also the more evolutionary conserved regions in the MSA. A Consurf[12,13,14] analysis for the 104 amino acids, to identify functional regions, also pointed at the same region (see Figure S8B). These analyses lead us to hypothesise that two small structured regions (residues 472-482 and 456-461) might be important for binding. Next, we used MD simulations with mixed aqueous/organic solvents (MDmix)[15,16] to identify binding hot spots and assess the druggability of the section of KRT1 structure that was deemed important for binding (residues 454-484). The analysis revealed that, in spite of the highly dynamic and solvent-exposed characteristics of a sequence that lacks tertiary structure, it displays 5 different binding hotspots with very high affinity for hydrophobic groups (Figure S9), consistent with the hypothesis that this region of KRT1 acts as a binding site.

Conformational analysis of the cyclopeptides

The structure of peptide **2** was created in MOE[17] using the Protein Builder panel. The chirality of the Lys residue was inverted using the Builder panel. In order to cyclise the peptide, first a soft restraint was created between the amino of the first residue and the carboxylic carbon of the last one. Minimisation brought these atoms nearby, then the residues were joined with the Protein Builder panel and the systems was minimised again. This was used as the initial conformation for molecular dynamics with Amber. For the labelled molecules **8** and **9**, Trp and Lys were replaced by the corresponding BODIPY-containing residues (named KPY and WPY

hereafter). Non-standard residues (norLeu, KPY, WPY) were parameterised using the RESP procedure[18] to assign partial charges. Atom types (providing the Lennard-Jones and bonded terms) were manually assigned from the Amber force-field. OFF files for these residues are available upon request. Finally, the tLeap program (part of Amber Tools) was used to embed the cyclic peptides into a cubic TIP3P water box spanning at least 20 Å in each direction and to generate Amber topology and coordinate files. 2000 steps of standard minimisation were carried out. Both the equilibration and production protocols were standard ones, using a Langevin thermostat with a collision frequency of 4 ps-1 and the cut-off for non-bonded interactions was set to 9 Å. All bonds are constrained using SHAKE and an integration step of 2 fs was used. Periodic boundary conditions and Ewald sums (grid spacing of 1 Å) were used to treat long range electrostatic interactions. The systems were heated from 100 K to 300 K in 800 ps in the NVT ensemble, followed by 1 ns of equilibration at 300 K in the NPT ensemble. Then, we ran a 1 microsecond production run for each molecule, saving coordinates in the netcdf trajectory files. All the simulations were performed with AMBER18[19] adapted for running in graphics processing unit (GPUs) and executed at the Barcelona Supercomputing Centre using NVIDIA V100 GPUs. Trajectory analysis was carried out with the cpptraj module, using the dbscan algorithm to cluster the conformations using the RMS of non-hydrogen atoms as distance metric and an epsilon value of 1.5 Å. The populations of the top 5 clusters are shown in Table S1. Their structures can be obtained upon request from the authors.

Conformational analysis of the protein

Three replicas of MD in water were performed using Amber ff99SB[20] force field for protein KRT1. TP3P box was chosen for solvation of the system, with a volume of 3406570 A^3. Three Na^+ ions were necessary to neutralise the system; the system was then minimised and equilibrated at the default temperature of 300 K.

Hot spot identification with MDmix

As the putative binding site (residues 454-484) of the protein preserves its secondary structure, and in order to obtain converged and accurate MDmix results, we applied soft restraints (harmonic restraints with force constant of 0.01kcal/mol·$Å^2$), to the non-hydrogen atoms of the protein, as recommended.[21] As shown in the RMSF and RMSD plots, (Figure S10) the protein fluctuates but preserves its original structure. Three replicas of MD in Ethanol (20%) in water were performed for a shorter section (res. 454-484) of the protein, this time using MDmix. [15,16] The method identifies hydrophobic and hydrogen bond donor or acceptor, and these are key points for the most important protein-drug interactions. In fact, we found 5 different pharmacophoric points, along the protein, which were then using for Docking studies.

Sequence analysis

We run Consurf[12,13,14] webserver for the PDB structure, to find functional regions in the protein, using HMMER (Homolog Search Algorithm), using a number of iterations of 5. The algorithm found 150 sequences homologues to the protein over 9821 HMMER hits. The most conserved and thus the functional amino acids are located at the same regions, in the C-terminal of the protein. The MSA was done using Clustal Omega.[11]

Binding mode prediction

In order to determine a possible binding mode of the cyclopeptide, we ran docking simulations with rDock.[22] The usefulness of rDock for the specific problem of protein-peptide docking has been assessed very recently, with positive results.[23] For the cavity preparation we used the refence ligand method, as implemented in the rbcavity module. The coordinates of the binding hot spots identified in the previous section where used as reference, with a radius of 10 Å. This creates the cavity depicted in Figure S11, providing ample space for accommodating the cyclopeptides. As rDock considers the internal degrees of freedom explicitly, but treats ring systems as rigid bodies, we provided multiple structures of each cyclic peptide as input. In particular, we used the centroids of those clusters representing more than 2% of the population observed in the MD simulations. That is 3 structures for **2**, and 4 structures for **8** and **9** (see Table S1). We ran 100 independent docking calculations for each input ligand structure and sorted all results according to the intermolecular score. The top-scoring poses are shown in Figure S12. Note that the position of the hot spots or the binding mode of KRT10 was not used to direct the docking results. For this reason, the overlap of the predicted binding mode of **2** and **8** with the MDmix hot spots and the structural alignment with KRT10 provide confidence on the results.

4. Supplementary Figures and Tables

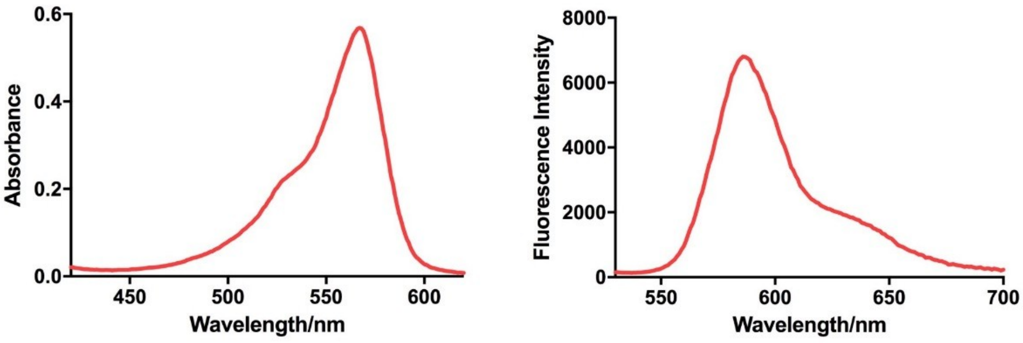

Figure S1. Absorbance (left) and emission (right) spectra of Trp(redBODIPY) (**1**) (10 μM) in EtOH. $\lambda_{exc.}$: 500 nm.

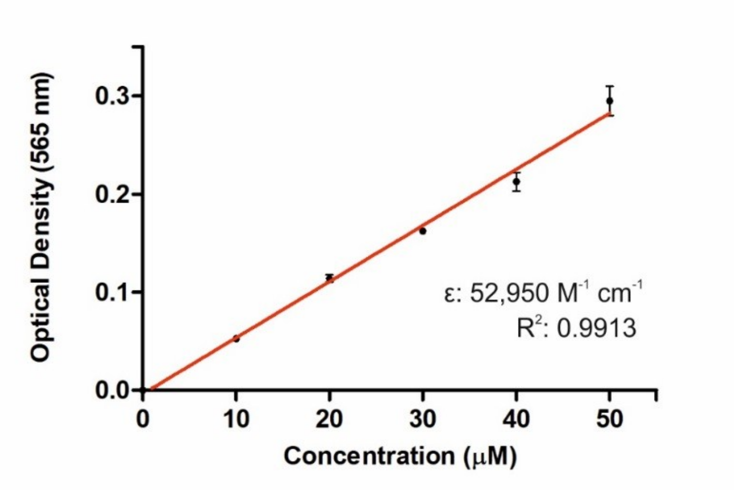

Figure S2. Determination of the extinction coefficient of Trp(redBODIPY) (1). Solutions of the amino acid at different concentrations were prepared in EtOH and their optical densities were measured at 565 nm in a NanoDrop 1000 spectrophotometer. Values are presented as means ± s.e.m. (n=3).

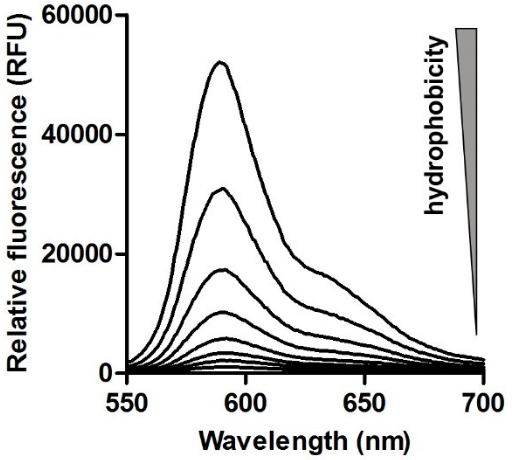

Figure S3. Fluorogenic response of Trp(redBODIPY) (1). Fluorescence spectra of Trp(redBODIPY) **(1)** (30 μM) were measured in suspensions of phosphatidylcholine-based liposomes of increasing hydrophobicity (from 0.5 mg mL^{-1} to 4 μg mL^{-1} in 2-fold serial dilutions). The fluorescence spectrum of the amino acid **1** in PBS is shown in black. $\lambda_{exc.}$: 520 nm.

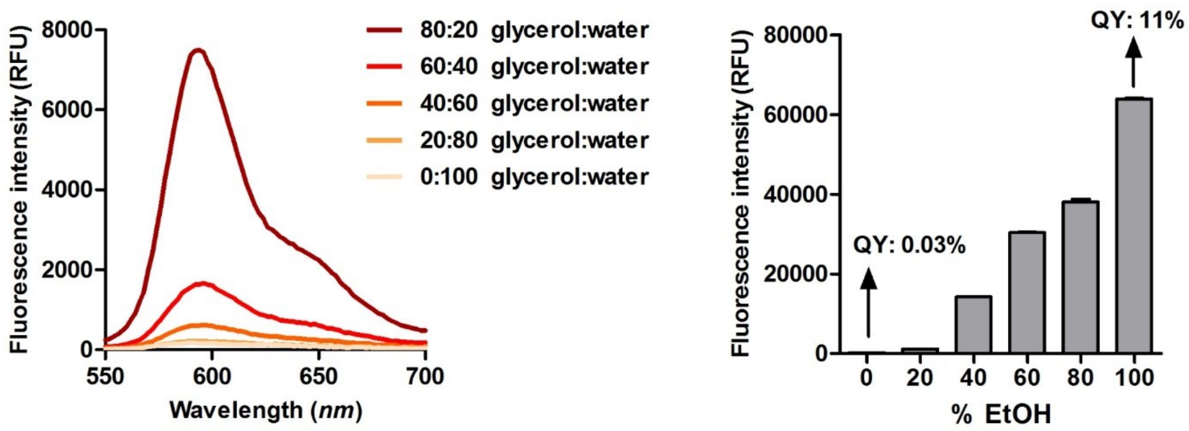

Figure S4. Spectral characterisation of Trp(redBODIPY) (1) in different solvent mixtures. Fluorescence spectra of Trp(redBODIPY) **(1)** were measured in glycerol-water mixtures (left) and in ethanol-water mixtures (right). $\lambda_{exc.}$: 520 nm, $\lambda_{em.}$ (right plot): 590 nm.

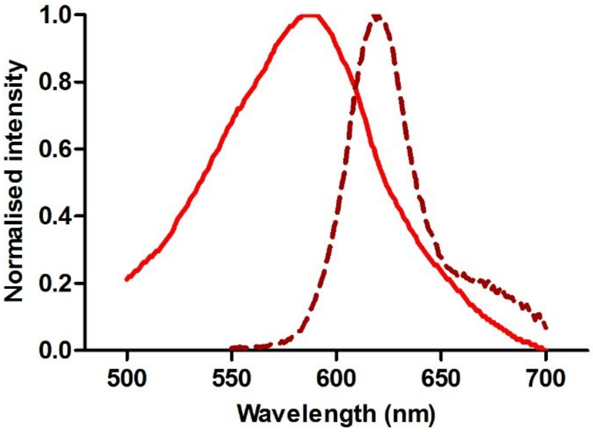

Figure S5. Absorbance and emission spectra of BODIPY TR-labelled peptide **9** (10 μM) in EtOH. Normalised absorbance (solid line) and fluorescence emission (dashed line, $\lambda_{exc.}$: 520 nm).

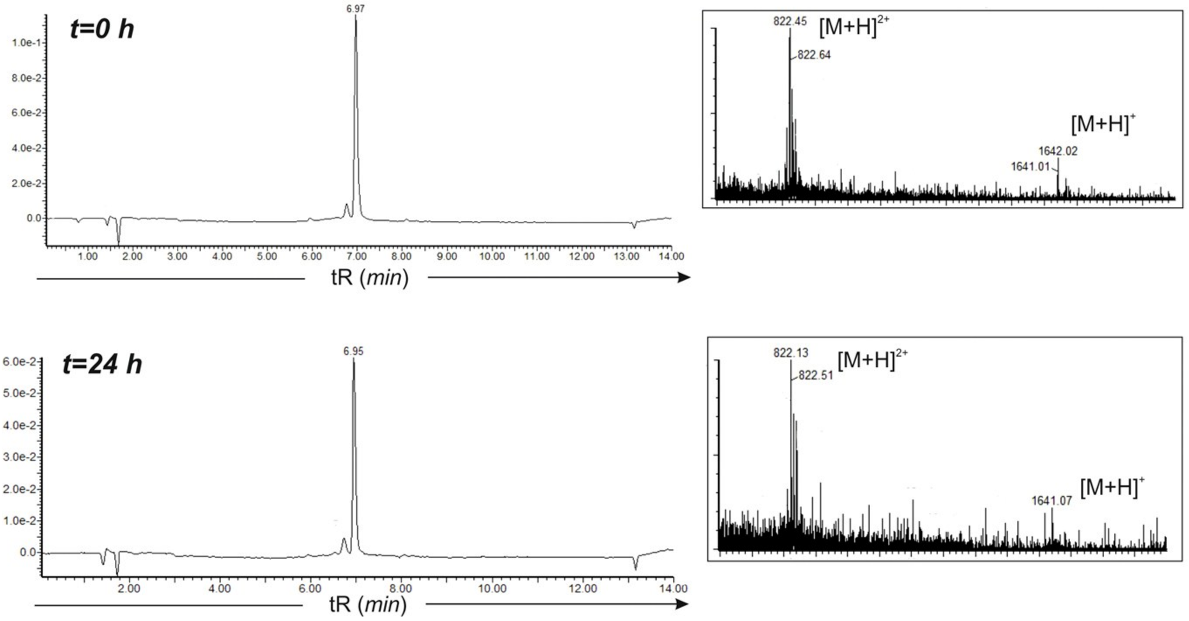

Figure S6. Proteolytic stability of peptide 8. HPLC-MS traces of Trp(redBODIPY)-labelled peptide **8** before and after 24 h treatment with a protease cocktail from *Streptomyces Griseus* at 37˚C (5 μg mL^{-1}, pH 7.1). Insets show the mass spectra (ESI+) of the corresponding main peaks in the HPLC chromatogram.

Table S1. Population of the top 5 clusters, as obtained from unrestricted MD simulations. The class 'Other' includes all conformations not included in any of the top 5 clusters.

	Population		
**Family**	**2 (unlabelled)**	**9 (KPY)**	**8 (WPY)**
**1**	68%	46%	64%
**2**	11%	7%	16%
**3**	5%	5%	4%
**4**	2%	4%	4%
**5**	2%	2%	1%
**Other**	13%	36%	11%

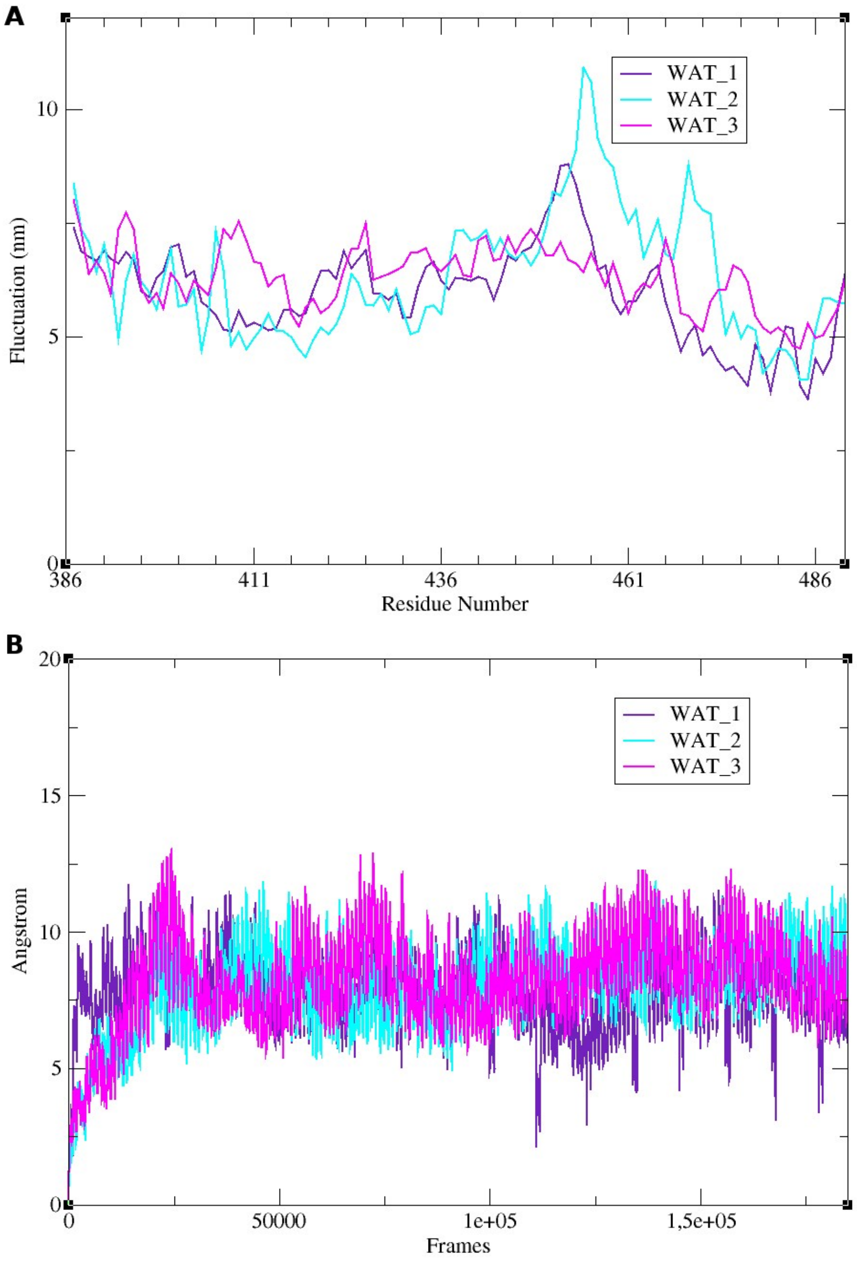

Figure S7. RMSF (A: top) and RMSD (B: bottom) graphs for the MD unrestrained simulations of KRT1 in water (3 replicas, 200 ns each).

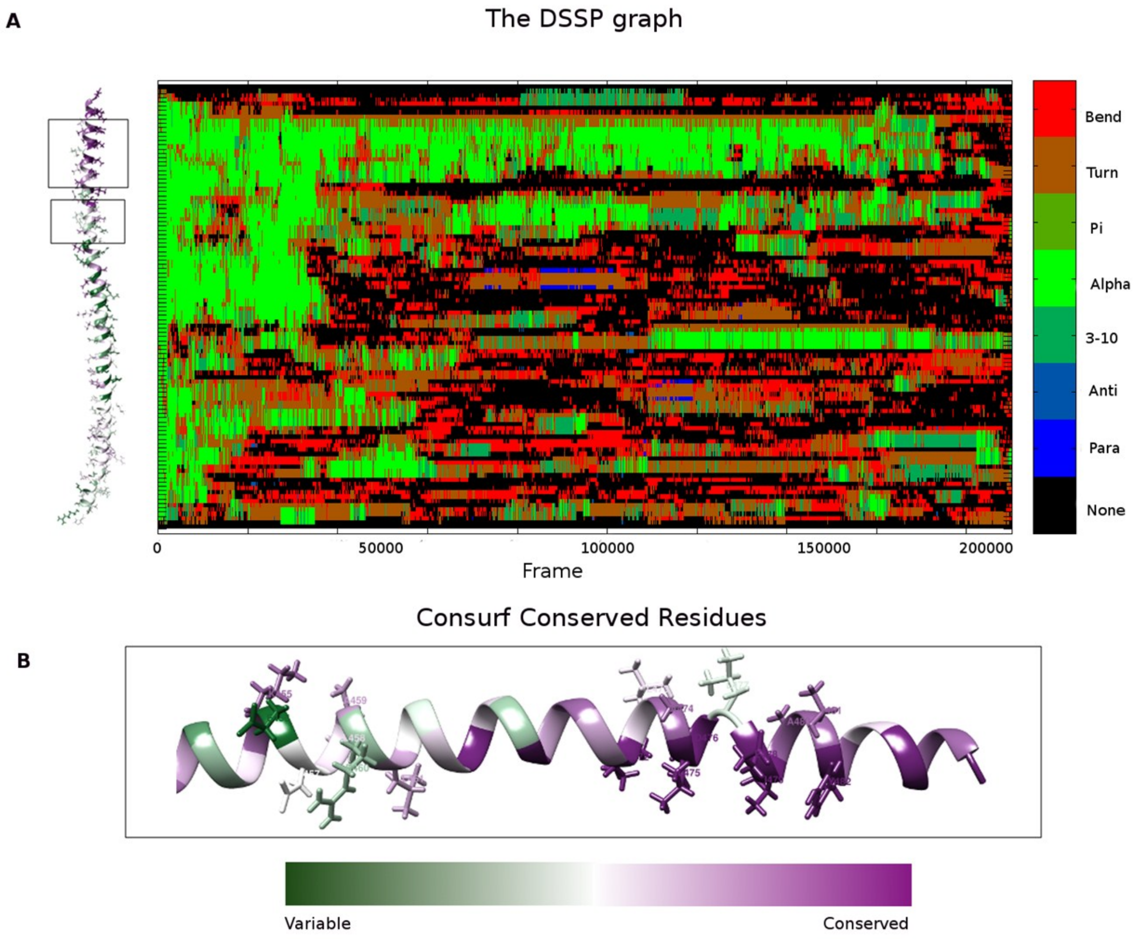

Figure S8. Structural and conservation analysis point at residues 456-461 and 472-482 as functionally important. A) The DSSP graph shows the evolution of the secondary structure of KRT1 (residues 386 to 489) during the MD simulations. The amino acid structure is represented versus the MD snapshots (frames). Residues 456-461 and 472-482 (highlighted with grey boxes) stay folded during the simulation, while the remaining residues lose their secondary structure. B) The Consurf conserved residue scheme: residues 456-461 and, particularly, 472-482 were the most conserved.

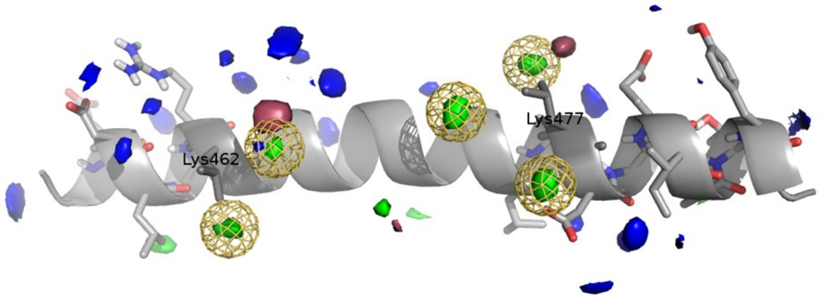

Figure S9. Binding hot spots identified with MDmix simulation. The yellow mesh and green surface indicate high density of ethanol methyl group; i.e. hydrophobic binding hot spots. Bourgogne surface indicates areas of high density of ethanol hydroxyl group; i.e. polar binding hot spots. Blue surface indicates areas of high water density; i.e areas that prefer to remain solvated.

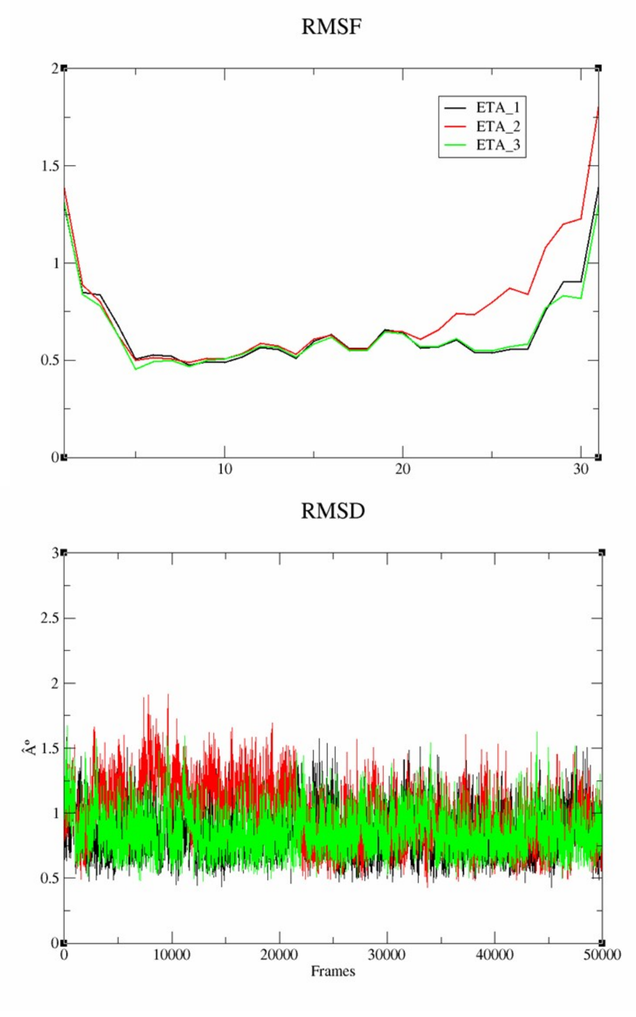

Figure S10. Mobility analysis of KRT1 in MD simulations. RMSF (top) and RMSD (bottom) for residues 454-484 of KRT1 with soft cartesian constraints (k=0.01kcal/mol·Å^2), solvated with a mixture of 20% ethanol in water (3 replicas, 50 ns each).

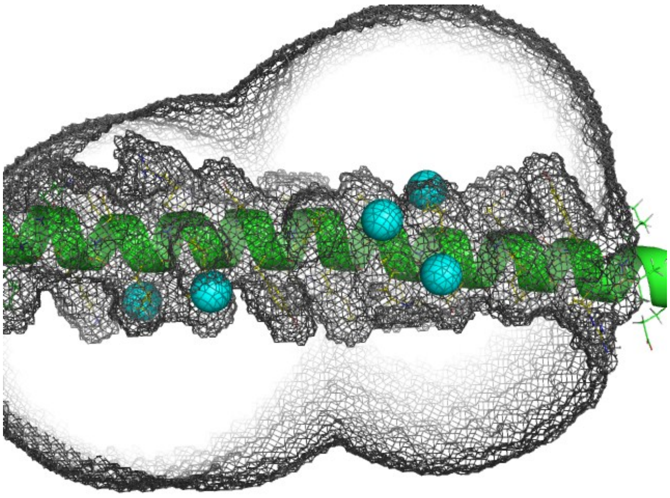

Figure S11. Cavity definition for docking calculations. The centre of the binding hot spots were identified with MDmix (cyan spheres) and used as reference coordinates. Any space within 10 Å of those centres that is not considered excluded volume (i.e. volume occupied by the KRT1 protein; green ribbon), is considered as part of the docking cavity (space inside the black mesh).

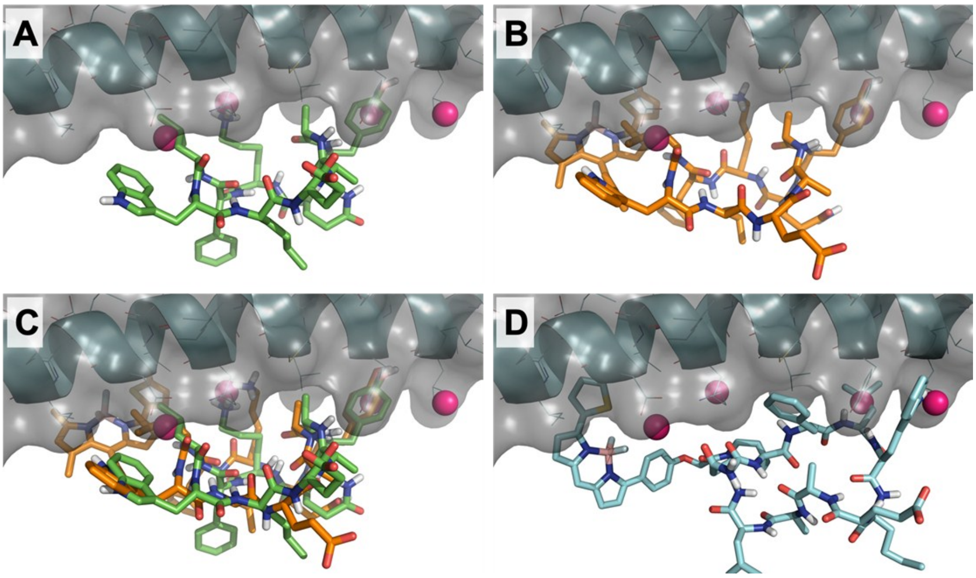

Figure S12. Top-scoring binding modes of the cyclopeptides to KRT1 (grey surface with cyan cartoon) as predicted by docking. A) The predicted binding mode of peptide **2** (green) overlaps with three of the binding hot spots identified with MDmix (pink spheres). B) Peptide **8** (orange) is predicted to bind in the same way as **2**. C) Superimposition of the predicted binding modes for **2** and **8**. D) Compound **9** does not recapitulate the same binding mode, and the predicted binding mode (cyan) scores worse that the others.

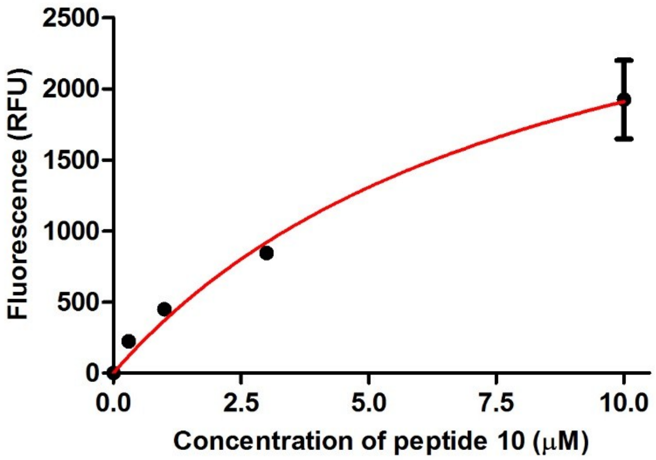

Figure S13. Flow cytometric analysis of MDA-MB-231 (KRT1+ cells) upon incubation with increasing concentrations of the BODIPY TR-labelled peptide **9**. Values are presented as means ± s.d. (n=3). Kd value = 8.6 μM (R^2: 0.973).

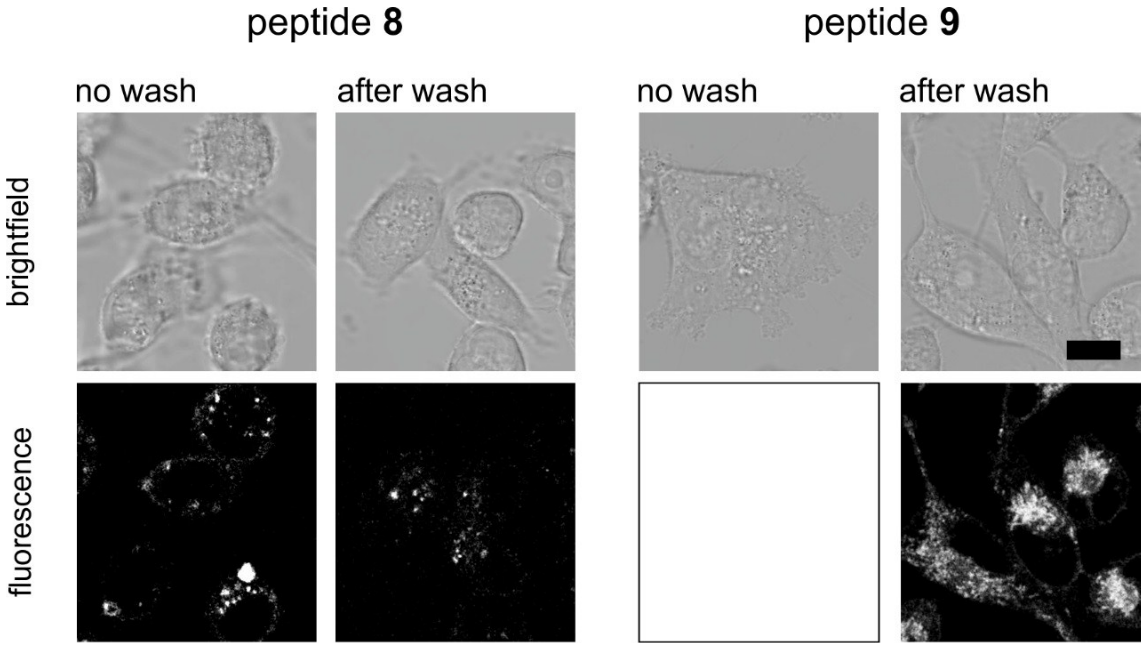

Figure S14. Brightfield and grayscale fluorescence microscope images of MDA-MB-231 cells upon incubation with the peptides **8** or **9** (both at 1 μM) and before and after washing with PBS. The no-wash fluorescence image with peptide **9** shows no distinguishable features due to the always-on character of the fluorophore. Scale bar: 10 μm.

<u>**5. Experimental Protocols for Biological Assays**</u>

Spectroscopic characterisation

Spectral properties were measured in a BioTek Cytation 3 spectrophotometer. Absorbance and emission spectra were determined in the range of 400–700 nm (every 1 nm) at the indicated concentrations. Environmental sensitivity was measured by comparing the emission in PBS or in presence of phosphatidylcholine: cholesterol (7:1) liposomes (3.75 mg mL^{-1} PC). Fluorescence emission was determined on a 384-well plate using a BioTek Cytation 3 spectrophotometer as indicated (excitation at 520 nm, 530-700 nm emission range, every 1 nm).

Protease stability assays

The chemical stability of the peptides was assessed in the presence of Protease XIV (from *Streptomyces Griseus*) in PBS-citrate buffer (pH 7.1) at 37ºC. First, a stock of 5 µg mL^{-1} of the protease cocktail in the buffer was prepared. Initially, a concentrated 1 mg mL^{-1} solution was obtained by weighing 1.1 mg of the protease and dissolving it in 1.1 mL of buffer, and afterwards diluting it down to the desired 5 µg mL^{-1} stock. Then, 5 µL of the peptides (5 mM in DMSO) were diluted with 45 µL of the protease cocktail to obtain a final peptide concentration of 500 µM. Peptides were vortexed, covered in aluminium foil and shaken at 37°C for 2 h or 24 h. Aliquots (25 µL) were taken, proteins were precipitated by microcentrifugation at 4°C, and supernatants were collected for HPLC-MS with detection at 280 nm.

Cell viability assays

Cell viability of HeLa cells was determined using the TACS® MTT Cell Proliferation assay (Trevigen). HeLa cells were plated on 96-well plates (10,000 cells/well) the day before the experiment and incubated overnight at 37 °C in 5% CO_2, reaching 90-95% confluence on the day of the experiment. Cells were incubated with or without peptide **8** at the indicated concentrations for 2 h at 37 °C. Afterwards, cells were washed and treated according to manufacturer's instructions. Cell viability was normalised to the proliferation of cells without addition of the peptide.

Cell culture and transfection

MDA-MB-231 and HUVEC cells were obtained from LGC Standards and cultured in Dulbecco's modified Eagle's medium (DMEM) supplemented with 10% FBS, 100 U mL^{-1} penicillin and 0.1 mg mL^{-1} streptomycin at 37°C and with 5% CO_2.

Ethical statement

This study was performed in strict accordance with the guidelines on the United Kingdom Animals Act 1986 for the care and use of laboratory animals. The work was approved by the Home Office (Animals in Science Regulation Unit) and performed under the local rules and guidance of the Animal Welfare and Ethical Review Body at The University of Edinburgh (Edinburgh, UK), which is a designated User and Supplying Establishment under ASPA (1986) with PEL Number 60/6205.

Fluorescence confocal microscopy

Cells were plated on glass chamber slides Lab-Tek™ II (Nunc) the day before the experiments to reach confluency around 70-90%. Cells were incubated with the fluorescent peptides (1 μM) at 37 °C and imaged under a Leica SP8 fluorescence confocal microscope equipped with a live-cell imaging stage. Fluorescence and brightfield images were acquired using a 40X oil objective. Fluorophores were excited with 405 nm, 488 nm or 561 nm lasers. Images were analysed and processed with ImageJ.

Ex vivo imaging of tumours. Athymic nude mice were bought from Charles River and 2×10^5 MDA-MB-231 cells were injected in each flank to develop subcutaneous tumours. After 14 days, subcutaneous carcinomas were harvested from mice, fixed overnight in ice-cold paraformaldehyde (4% in PBS) and transferred into 70% ethanol. Carcinomas were then embedded into paraffin and sections of 5 μm thickness prepared. Paraffin was removed in xylene 5 min (× 2), followed by rehydration with EtOH [100% (× 2), 95%, 80% and 70%] each for 20 seconds and finally in distilled water. Antigen retrieval was performed with 1X citric buffer, pH 6. After washing with PBS, slides were blocked using 5% BSA for 30 min at r.t. and stained with peptide **8** (5 μM) in 20 mM HEPES, 140 mM NaCl, 0.1% BSA and 1% DMSO for 1 h at 37 ºC. Prior to staining with anti-Iba1 antibody (abcam, ab178846, 1/500 dilution) in 5% BSA at 4 ºC in a humidified chamber overnight, samples were blocked for 30 min with 5% BSA at r.t. Secondary antibody staining was performed with goat anti-rabbit IgG (H+L) AF488 (ThermoFisher Scientific, A11034) for 2 h in a humidified chamber at r.t. Nuclear material was stained with 1 $\mu g\ mL^{-1}$ DAPI in dH_2O for 5 min at r.t. Slides were mounted using VectaShield Hard Set mounting medium and images were acquired with the Zeiss Axio Scan.Z1 microscope using the Zeiss Zen2 software.

Flow cytometry analysis

MDA-MB-231 cells (about 3×10^5 cells) were incubated with the peptides at the indicated concentrations for 30 min at 37 °C in FACS buffer (20 mM HEPES, 140 mM NaCl, 0.1% BSA). Samples were spun at 300 g for 2 min at room temperature, and the pellets were resuspended in FACS buffer prior to flow cytometry. Fluorescence emission was acquired on a 5L LSR flow

cytometer. Excitation sources/emission filters used: 561 nm/ 600 nm. Data was analysed with the FlowJo software.

6. Supplementary References

1. W. C. Chan and P. D. White, *Fmoc solid phase peptide synthesis*. 376 (Oxford University Press, New York, 2000).

2. C. N. Johnson, G. Stemp, N. Anand, S. C. Stephen and T. Gallagher, *Synlett,* **1998**, *9*, 1025.

3. I. Hasan, E. R. Marinelli, L.-C. C. Lin, F. W. Fowler and A. B. Levy, *J. Org. Chem.*, **1981**, *46*, 157.

4. C.-Q. Wang, L. Ye, C. Feng and T.-P. Loh, *J. Am. Chem. Soc.*, **2017**, *139*, 1762.

5. A. Bessette, T. Auvray, D. Désilets and G. S. Hanan, *Dalton Trans.,* **2016**, *45*, 7589.

6. L. Mendive-Tapia, C. Zhao, A. R. Akram, S. Preciado, F. Albericio, M. Lee, A. Serrels, N. Kielland, N. D. Read, R. Lavilla and M. Vendrell, *Nat. Commun*., **2016**, *7*, 10940.

7. C. G. Bunick, L. M. Milstone, *J. Invest. Dermatol*., **2017**, *137*, 142.

8. S. A. Eldirany, M. Ho, A. J. Hinbest, I. B. Lomakin, C. G. Bunick, *EMBO J*., **2019**, *38*, e100741

9. R. Soudy, H. Etayash, K. Bahadorani, A. Lavasanifar, K. Kaur, *Mol. Pharm.,* **2017**, *14*, 593.

10. W. Kabsch, C. Sander, *Biopolymers*, **1983**, *22*, 2577.

11. F. Sievers, A. Wilm, D. Dineen, T. J. Gibson, K. Karplus, W. Li, R. Lopez, H. McWilliam, M. Remmert, J. Söding, J. D. Thompson, D. G. Higgins, *Mol. Syst. Biol.* **2011**, *7*, 539.

12. H. Ashkenazy, S. Abadi, E. Martz, O. Chay, I. Mayrose, T. Pupko, N. Ben-Tal, *Nucl. Acids Res.,* **2016**; doi: 10.1093/nar/gkw408; PMID: 27166375.

13. G. Celniker, G. Nimrod, H. Ashkenazy, F. Glaser, E. Martz, I. Mayrose, T. Pupko, N. Ben-Tal, *Isr. J. Chem.* **2013** March 10, doi: 10.1002/ijch.201200096.

14. H. Ashkenazy, E. Erez, E. Martz, T. Pupko, N. Ben-Tal. *Nucl. Acids Res.* **2010**; doi: 10.1093/nar/gkq399.

15. J. Seco, F. J. Luque, X. Barril, *J. Med. Chem.* **2009**, *52*, 2363.

16. D. Alvarez-Garcia, X. Barril, *J. Med. Chem.* **2014**, *57*, 8530.

17. Molecular Operating Environment (MOE). Chemical Computing Group ULC: 1010 Sherbooke St. West, Suite #910, Montreal, QC, Canada, H3A 2R7, **2018**.

18. C. I. Bayly, P. Cieplak, W. Cornell, P. A. Kollman, *J. Phys. Chem.* **1993**, *97*, 10269.

19. T. S. Lee, D.S. Cerutti, D. Mermelstein, C. Lin, S. LeGrand, T. J Giese, A. Roitberg, D. A. Case, R. C. Walker, D. M. York, *J. Chem. Inf. Model.* **2018**, *58*, 2043.

20. J.A. Maier, C. Martinez, K. Kasavajhala, L. Wickstrom, K.E. Hauser, C. Simmerling, *J. Chem. Theory Comput.* **2015**, *11*, 3696.

21. D. Alvarez-Garcia, X. Barril, *J. Chem. Theory Comput.* **2014**, *10*, 2608.

22. S. Ruiz-Carmona, D. Alvarez-Garcia, N. Foloppe, A. B. Garmendia-Doval, S. Juhos, P. Schmidtke, X. Barril, Roderick E. Hubbard, S. D. Morley, *PLoS Comput. Biol*. **2014**, *10*, e1003571.

23. D. Soler, Y. Westermaier, R. Soliva, *J. Comput. Aided. Mol. Des.* **2019**, *33*, 613.
